# Dam-dependent epigenetic memory regulates prophage reintegration in *Salmonella*

**DOI:** 10.1093/nar/gkaf951

**Published:** 2025-09-23

**Authors:** Jihye Yang, Yongjun Son, Jinwon Park, Woojun Park

**Affiliations:** Laboratory of Molecular Environmental Microbiology, Department of Environmental Science and Ecological Engineering, Korea University, Seoul 02841, Republic of Korea; Laboratory of Molecular Environmental Microbiology, Department of Environmental Science and Ecological Engineering, Korea University, Seoul 02841, Republic of Korea; Laboratory of Molecular Environmental Microbiology, Department of Environmental Science and Ecological Engineering, Korea University, Seoul 02841, Republic of Korea; Laboratory of Molecular Environmental Microbiology, Department of Environmental Science and Ecological Engineering, Korea University, Seoul 02841, Republic of Korea

## Abstract

Bacterial epigenetics has emerged as a critical mechanism for regulating gene expression in response to environmental cues, yet whether such modifications persist beyond initial stress remains unresolved. Here, we uncover an epigenetic memory system in *Salmonella enterica* that facilitates prophage reintegration during infection. Using an *in vitro* model mimicking stages of the *Salmonella* infection cycle, including the *Salmonella*-containing vacuole (SCV), we found that DNA adenine methyltransferase (Dam) plays stage-specific roles in adaptation and survival of *Salmonella* cells. Early during SCV-like stress, oxidative stress contributes to excision of the cryptic prophage ST64B. However, at later infection stages, levels of excised phage DNA decline as global methylation increases, notably at the promoter of *intA*, a prophage integrase encoded by *Salmonella*. This methylation persists after stress removal and maintains active *intA* transcription, establishing a form of epigenetic memory. Functional assays revealed that *intA* expression is required for efficient ST64B reintegration and that this process depends on methylation at a critical GATC site within the promoter. Mechanistically, we show that SCV stress disrupts binding of the integration host factor, a repressor of *intA*, thereby enabling Dam-mediated methylation. Sustained *intA* activation stabilizes prophage reintegration, highlighting stress-responsive epigenetic control important for adaptation during *Salmonella* pathogenesis.

## Graphical Abstract

**Graphical Abstract:**
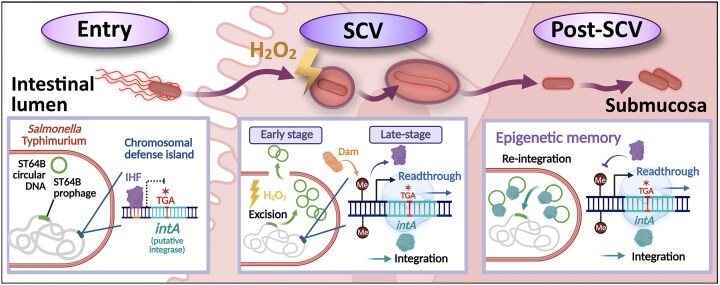

## Introduction

*Salmonella enterica* serovar Typhimurium is a facultative intracellular pathogen and a leading cause of foodborne gastroenteritis. To persist within the host, this bacterium undergoes extensive physiological reprogramming in response to various environmental factors [1]. A key feature of its intracellular lifestyle is the development of the *Salmonella*-containing vacuole (SCV), a host-derived membrane compartment that transitions from an entry-associated structure to a replicative niche [2]. SCV formation depends on the coordinated activity of the type III secretion system (T3SS), encoded by *Salmonella* pathogenicity islands 1 and 2 (SPI-1 and SPI-2, respectively). SPI-1 effectors, such as SipA, SopE, and SipC, manipulate host actin dynamics to induce membrane ruffling and promote bacterial uptake by epithelial cells [3, 4]. In contrast, within macrophages, SPI-2 effectors, such as SseF and SseG, alter SCV membrane trafficking by targeting Rab GTPases and preventing fusion with lysosomes, thereby facilitating intracellular replication [5]. As the SCV matures, *Salmonella* is exposed to diverse stressors, including nutrient limitation, iron restriction, and host-derived reactive oxygen species (ROS) [6, 7].

Stress conditions necessitate adaptive mechanisms. The key DNA adenine methyltransferase in bacteria, Dam, has been shown to enhance bacterial fitness under oxidative stress [8]. Accordingly, the loss of *dam* impairs the survival of *S*. Typhimurium in intracellular niches [8]. Prolonged nutrient deprivation increases methylation at stress-related loci in *Escherichia coli*, contributing to altered gene expression under starvation [9]. In *Mycobacterium tuberculosis*, MamA-mediated DNA methylation promotes survival under hypoxic conditions by specifically regulating promoters of genes such as the Mg^2+^ transporter *corA* and the global stress response regulator *whiB7* [10]. Together, these findings indicate that dynamic methylation changes may enable *Salmonella* to fine-tune gene expression programs that support its adaptation and survival within the SCV, but systematic study remains elusive. In eukaryotes, environmental stress can trigger epigenetic modifications that are stably inherited across generations [11]. For example, in *Arabidopsis thaliana*, cold exposure leads to heritable DNA methylation at the *NMR19-4* locus, modulating chlorophyll degradation during leaf senescence [12]. Even partial methylation of CpG sites (∼10%) can influence transcriptional memory by reshaping chromatin architecture [13–15]. In bacteria, although classical chromatin-based mechanisms are absent, epigenetic regulation can occur through stable, heritable changes, such as DNA methylation, that persist across cell divisions without altering the underlying DNA sequence [16]. Although Dam lacks a dedicated replication-coupled maintenance complex like DNMT1, it displays a strong preference for hemimethylated DNA [17]. As the primary methyltransferase in *Salmonella*, this substrate specificity enables Dam to efficiently restore full methylation following DNA replication, functionally analogous to maintenance methylation in eukaryotes. However, the persistence of DNA methylation in prokaryotes under stress conditions remains poorly understood. Unlike eukaryotes, bacteria lack active demethylation enzymes. Instead, they rely on maintenance MTases to replicate methylation patterns during DNA synthesis, limiting their ability to initiate *de novo* methylation changes [18]. Bacterial epigenetics has primarily focused on phase variation, but the stability of methylation marks in fluctuating environments remains unclear [19–21]. Hence, it is crucial to elucidate how *Salmonella* converts intracellular stress into epigenetic memory.

A parallel dimension of epigenetic regulation involves cryptic prophages, viral elements embedded in the bacterial genome that are typically latent but can be reactivated under stress conditions, such as oxidative damage or nutrient deprivation. Within SCV-like environments, *Salmonella* prophages, such as Gifsy-1 and ST64B, are excised in response to ROS and nutrient limitation. This process is driven by the induction of excisionase and other recombination directionality factors [22]. Some prophages, such as Gifsy-2, carry genes encoding virulence factors, e.g. *sopE* and *sodC*, which contribute to pathogenesis by enhancing host membrane ruffling and neutralizing ROS, respectively [23]. Similarly, in *E. coli*, stress-induced activation of the Qin prophage induces the expression of DicF small RNA and DicB protein, both of which inhibit cell division [24]. Genome-wide methylation profiling has revealed that stress-responsive prophages, such as Qin, exhibit significantly lower methylation levels (95.1%) than the genome average (97%) (*P* = 2.9 × 10**^−^**^6^), suggesting a functional link between local hypomethylation and prophage gene activation [25]. Building on these insights, our study proposes that dynamic DNA methylation and integrase-centered regulation at a conserved chromosomal defense island in *S. enterica* serovar Typhimurium 14028s coordinate prophage stability. We further suggest that this regulatory system facilitates the conversion of host-derived stress signals into sustained epigenetic adaptive responses during infection.

## Materials and methods

### Bacterial culture conditions and infection cycle–mimicking time-course experiments

**Figure 1.**
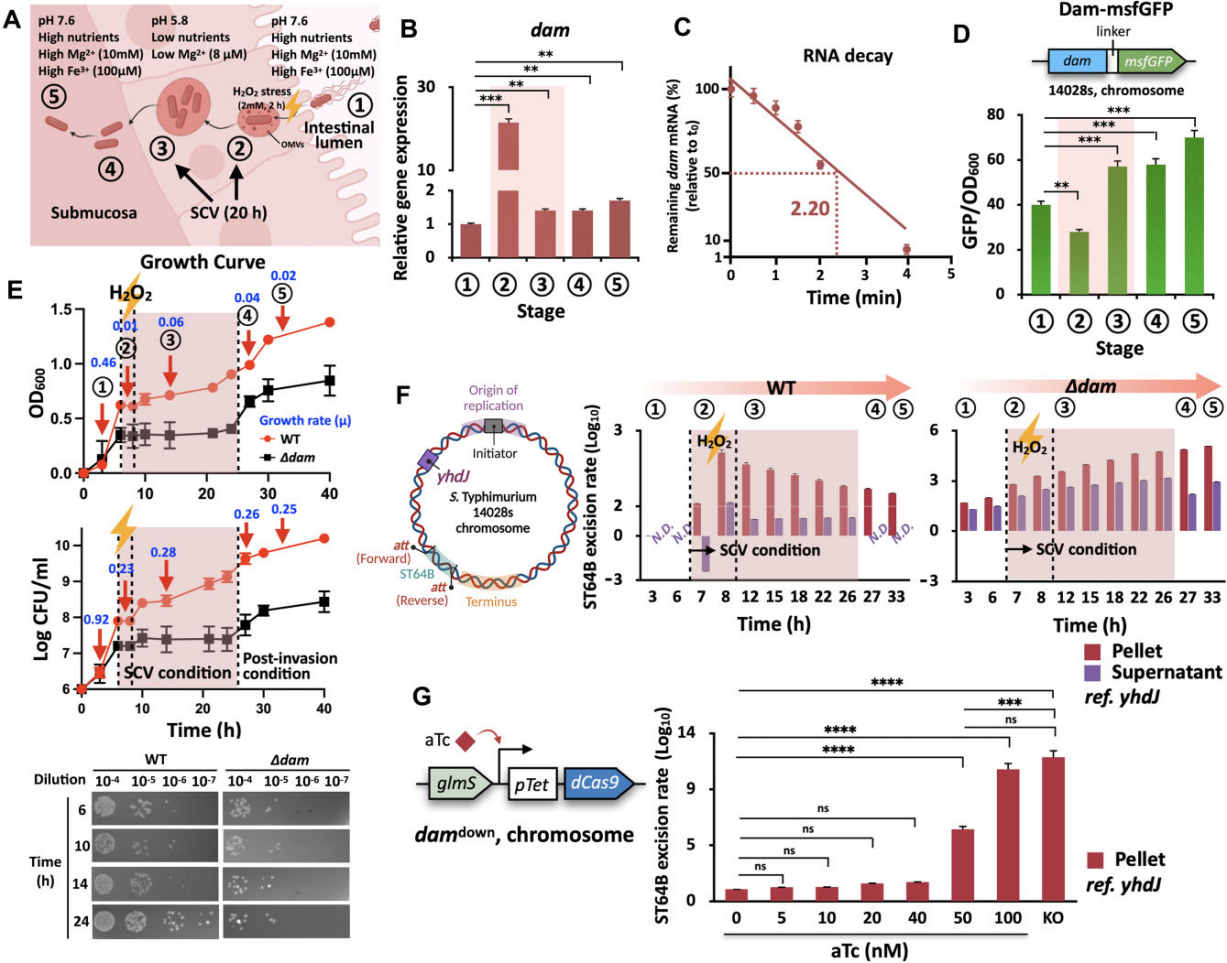
Stage-specific roles of Dam in SCV adaptation and prophage dynamics. (**A**) *In vitro* infection-mimicking model of *Salmonella* pathogenesis. Five stages simulating *Salmonella* transition from the intestinal lumen (stage 1: LB medium) to the SCV (stage 2: LPM + H_2_O_2_; stage 3: LPM) and the post-SCV recovery phase (stages 4 and 5: LB medium). (**B**) *dam* transcription level evaluated by qRT-PCR. (**C**) mRNA decay of *dam* at stage 2 (half-life = 2.20 min). (**D**) Intracellular protein level based on Dam–msfGFP fluorescence. (**E**) Growth curves of WT and *Δdam* strains during the *in vitro* infection-mimicking condition. Diluted cultures were spotted on LB agar plates (bottom panel). (**F**) ST64B excision rates quantified by qPCR. Detected *attP* of ST64B was normalized by *ydhJ* (left panel). (**G**) CRISPRi-mediated *dam* silencing. Chromosomal dCas9 was induced by aTc (left panel). All experiments were performed in triplicate. The *P*-values are designated by asterisks (***P < 0.001 and ****P < 0.0001 based on Student’s *t*-test, two-tailed; ns, not specific).

For routine culture and *in vitro* experiments, *Salmonella* cells were grown in 50 ml of either lysogeny broth (LB; pH 7.6, 10 mM Mg^2+^, 100 μM Fe^3;+^; used for stages 1, 4, and 5) or low pH/Mg^2+^ medium (LPM; pH 5.8, 8 μM Mg^2+^; used for stages 2 and 3) at 37 °C without shaking, as previously described [26, 27]. For infection cycle–mimicking time-course experiments, overnight LB cultures were diluted 1:100 into fresh LB and grown to an OD_600_ of ∼0.5. Cultures were then diluted to 10^6^ CFU/ml and incubated in LB for 6 h at 37 °C (stage 1). Cells were harvested by centrifugation (4000 × *g*, 10 min, 25 °C), resuspended in 50 ml of LPM medium supplemented with 2 mM H_2_O_2_, and incubated at 37 °C without shaking for 2 h (stage 2; 1 h post-transition). For stage 3, cells were pelleted again and resuspended in fresh LPM medium lacking H_2_O_2_, followed by incubation at 37 °C for 18 h. To initiate stages 4 and 5, cells were transferred back to LB medium by centrifugation and resuspension. Cultures reached stage 4 after 1 h and stage 5 after 6 h of incubation at 37 °C. Where appropriate, antibiotic selection was applied using chloramphenicol (25 μg/ml), kanamycin (100 μg/ml), or gentamicin (25 μg/ml) in LB. Growth curves were generated by monitoring both optical density and CFU counts. For OD_600_-based measurements, values were recorded at each indicated time point using a spectrophotometer with 1 cm path-length cuvettes (Fig. 1E). In parallel, CFU counts were determined by serially diluting culture aliquots in phosphate-buffered saline (PBS), plating on LB agar, and incubating overnight at 37°C. All measurements were performed in biological triplicate using independently cultured samples.

### Validation of SCV-mimic condition using *P_sipA_*-sfGFP and *P_ssaC_*-sfGFP reporter strains

To monitor infection-stage-specific expression of SPI-1 and SPI-2 genes, we chromosomally replaced sipA and ssaC with cognate promoter-driven super-folder GFP (sfGFP) reporters (*P_sipA_*-sfGFP and *P_ssaC_*-sfGFP) using λ-Red recombination [28–30]. At each defined stage of the infection cycle, 1 ml of culture was harvested by centrifugation at 20 000 × *g* for 1 min at 4 °C and fixed in 4% paraformaldehyde. GFP fluorescence (excitation/emission: 488/510 nm) from each reporter strain was visualized using confocal laser scanning microscopy (CLSM; LSM 700, Carl Zeiss, Germany).

### Mutant construction

All strains, plasmids, and oligonucleotides used in this study are listed in Supplementary Tables S3 and S4. Chromosomal deletions, insertions, and point mutations were performed using the λ-Red recombination method. Polymerase chain reaction (PCR) products containing the KM or CM cassette were used to replace the target gene or were integrated into bacteria containing pKD46, as described previously [30]. Insertion was performed using PCR products containing the msfGFP or FLAG sequence. Point mutation was introduced with a primer containing the A–T mutation at the GATC site in the *intA* upstream region.

Subsequently, the KM cassette was removed through recombination at FRT sites using FLP recombinase encoded on pCP20. All deletions and mutations were verified by PCR, followed by sequencing using primers flanking the cassette or localized sequencing at the mutation site.

### RNA analyses

Total RNA from each target region in *S*. Typhimurium 14028s cells was extracted using the AccuPrep^®^ Bacterial RNA Extraction Kit (Bioneer, Republic of Korea), according to the manufacturer’s guidelines. The concentrations of the total recovered RNA were measured using a NanoPhotometer^®^ N60 (Implen, USA), and complementary DNA (cDNA) was then synthesized from the total extracted RNA (1 μg each). Residual DNA was removed using DNase I (Thermo Fisher Scientific, USA). To verify the absence of genomic DNA in every RNA sample, the reverse transcription (RT) reaction was performed in the presence and absence of RevertAid reverse transcriptase (Thermo Fisher Scientific, USA). The obtained cDNA was purified using a PCR purification kit (GeneAll, Republic of Korea), and 1 μl of the cDNA was used for performing PCR using AccuPower Taq PCR PreMix (Bioneer, Republic of Korea). The qRT-PCR assay was performed using the primers listed in Supplementary Table S4. The relative expression level of each gene was normalized to that of the 16S rDNA gene and analyzed using the QuantStudio 3 Real-Time PCR System (Thermo Fisher Scientific, USA). All quantitative Real-Time PCRqRT-PCR) procedures were performed in triplicate from at least three independent cultures.

### RNA stability assay

The half-life of *dam* mRNA was measured during stage 2 using a previously described protocol with minor modifications [31]. Cells were cultured until stage 2 (60 minutes after transition to LPM medium containing H_2_O_2_), at which point transcription was halted by the addition of rifampicin to a final concentration of 500 μg/ml, followed by shaking for 10 s. Cultures were maintained at 37 °C, and 1 ml aliquots were collected at 30, 60, 90, and 120 s, as well as 4 min after rifampicin addition. Each sample was immediately mixed with 5% phenol in 95% ethanol, flash-frozen in liquid nitrogen, and stored at –80 °C until RNA extraction. Total RNA was isolated, and dam transcript levels were quantified by qRT-PCR. Messenger RNA (mRNA) half-life was determined from the decay curve, based on three independent biological replicates.

### CRISPR interference

The schematic overview of constructing an anhydrotetracycline (aTc)-inducible CRISPR interference (CRISPRi) system in *S*. Typhimurium is provided in Supplementary Fig. S3. The mini-Tn7 element of the plasmid pYDE009, which contains an aTc-inducible CRISPRi module, was introduced into the 14028s strain harboring pBAD33 via four-parental mating [32]. The recipient 14028s strain with pBAD33, the *E. coli* DH5α strain (each containing the plasmids pYDE009 and pTNS3), and the helper strain *E. coli* HB101 containing the plasmid pRK2013 were grown O/N at 37°C with shaking (180 rpm). Each culture (1 ml) was combined to prepare a 4 ml of culture mix, and the sample was pelleted by centrifugation at 7600 × *g* for 1 min at 4°C. The cells were washed with 10 mM MgSO_4_ and subsequently washed with PBS. The mixture (500 μl) of the four strains was then plated onto a 0.45-μm-pore-size filter (diameter: 47 mm) in an LB plate and incubated for 4 h at 37°C. The resulting cells were washed off the filter with 1 ml of MgSO_4_, and 100 μl of the sample was spread onto an LB plate containing CM and GM for selection. The integration of dCas9 at the *att*Tn7 site was verified by PCR. The plasmid pBAD33 was cured through serial subculture without antibiotic selection. Counterselection was performed on LB plates with or without CM. For sgRNA expression, the spacer of *dam* was cloned into the pSGAb vector via Golden Gate Assembly and transformed into the chromosomal dCas9-harboring 14028s strain [33]. Then, aTc was gradually added to the CRISPRi system, and each induced sample was used for the subsequent ST64B excision assay.

### Western blot analysis

Protein samples from the crude extract of the 14028s strain with chromosomal C-terminal FLAG-tagged *intA* (*intA*_CT__FLAG) were diluted with a 5 × sample buffer (Elpis Biotech, Republic of Korea). The protein samples were then heated at 95°C for 5 min and loaded onto a 12% Sodium Dodecyl Sulfate-Polyacrylamide Gel Electrophoresis (SDS–PAGE) gel. After the completion of SDS–PAGE, the SDS gel was removed and immersed in a transfer buffer for 10 min. The Polyvinylidene Fluoride (PVDF) membrane (Thermo Fisher Scientific, USA) was activated by immersion in 100% methanol for 1 min. It was then rinsed with distilled water (DW) and stored in the transfer buffer [25 ml of 1 M Tris–HCl (pH 8.0), 14.4 g glycine, 200 ml of methanol, 1 g SDS, and DW up to a final volume of 1 L]. To manage the heat generated during this transfer, the tank was submerged in ice water. Moreover, the transfer was performed under the following conditions: voltage, 20 V; current, 400 mA; and duration, 150 min. Next, the protein-transferred membranes were incubated with the following primary antibodies: rabbit monoclonal anti-FLAG antibody (1:5000 dilution) and mouse monoclonal anti-GAPDH antibody (1:10 000 dilution). Goat anti-rabbit antibody (#170-6515; Bio-Rad; 1:10 000 dilution) and goat anti-mouse IgG (H + L) antibody conjugated with HRP (#31430; Thermo Fisher Scientific; 1:10 000 dilution) were used as secondary antibodies. The signal was generated by applying a western blot detection kit (WEST-SAVESTAR, Republic of Korea) to the membrane. The membrane was incubated at room temperature (RT) for 1 min, and immediate signal detection was performed using the iBright™ FL1500 Imaging System (Invitrogen, USA). Densitometric analysis was performed using ImageJ software (National Institutes of Health, USA). Three biological replicates were used to validate the data.

### Single-molecule real-time sequencing

DNA methylation analyses were performed by single-molecule real-time (SMRT, PacBio, USA) and high-throughput DNA *de novo* sequencing (DNAlink, Republic of Korea), as described previously [34]. Base modifications and methylated motifs were detected using the SMRT analysis portal following genome assembly. In brief, genomic DNA samples were extracted from each stage (1, 3, and 5) of the wild-type (WT) 14028s strain and used for sequencing and methylation analysis. Each genomic DNA sample was sheared into ≥15 kb using the Covaris g-TUBE and then purified using 0.45× AMPure XP magnetic beads. Each purified DNA fragment (500 ng) was utilized to generate the sequencing library using the SMRTbell Express Template Preparation Kit (ver. 3.0). To reveal genome-wide base modifications and identify *N*^6^-methyladenine (6mA) and *N*^4^-methylcytosine (4mC) residues in the corresponding methylation (recognition) motifs, the SMRTbell library was sequenced on PacBio Sequel II using SMRT Cell 8M (Pacific Biosciences, USA) and the Sequel Sequencing Kit 2.0 (Pacific Biosciences, USA) [35]. Reads were processed and mapped using the BLASR mapper (ver. 12.0) and the Pacific Biosciences SMRT Analysis pipeline as per the standard mapping protocol. Reads from the strains were mapped to the *S*. Typhimurium 14028s reference whole-genome sequence (accession number: CP001363). The pulse width and interpulse duration (IPD) ratio for each base, which are altered when the DNA polymerase copies past a modified nucleotide, were measured, and modification for each base was determined using an *in silico* control [36].

### Methylated DNA immunoprecipitation sequencing

Immunoprecipitation of 6mA fragments was performed as described previously [37]. Genomic DNA (gDNA) was extracted from WT and Δ*dam* strains at stages 1, 2, 3, and 5 and used for sequencing and methylation analysis. In brief, starting with 3 μg per sample, DNA was sheared with 30-s on/30-s off cycles using a Diagenode Bioruptor^®^ Pico (Diagenode, USA) for a total of 35 cycles at 4°C to generate fragments of ∼100–200 bp. A 3 μg DNA sample was taken for immunoprecipitation, and the remaining was set aside as an input control. For DNA immunoprecipitation (DIP), DNA was incubated O/N in 200 μl of 30 μg/μl bovine serum albumin (BSA) stock solution, 200 μl of 5× DIP buffer [0.5 ml of 1 M Tris–HCl (pH 7.4), 1.5 ml of 5 M NaCl, and 0.5 ml of 10% vol/vol Igepal CA-630], and water to a final volume of 1 ml in the presence of 2.5 μl of 1 μg/μl anti-6mA antibody stock (SYSY 202003). Magnetic protein A beads (Invitrogen) (100 μl/sample) were prepared by washing thrice in 1 ml of 1× DIP buffer and incubating O/N in 105 μl of 1× DIP buffer and 105 μl of BSA. Subsequently, 200 μl of the bead suspension was added to each sample, and the mixture was incubated for 90 min. The mixture was then washed four times with 1× DIP buffer, resuspended in 200 μl of DIP elution buffer (45 μl of 5× DIP buffer, 75 μl of 20 mM *N*^6^-methyladenosine 5′-monophosphate sodium salt stock, and 105 μl of water), and incubated at 42°C for 1 h by shaking at 1400 rpm. For precipitation, the supernatant was transferred to a new tube and mixed with 300 μl of water, 2 μl of GlycoBlue (Invitrogen), 50 μl of 3 M NaOAc, and 500 μl of isopropanol. The mixture was then frozen at −80°C, centrifuged for 30 min, washed twice in 70% ethanol, and resuspended in 1× TE. Libraries were constructed using the NEBNext^®^ Ultra^™^ DNA Library Prep Kit for Illumina (New England Biolabs, UK), according to the manufacturer’s instructions. In brief, chipped DNA was ligated with adaptors. After purification, PCR was performed using adaptor-ligated DNA and an index primer for multiplex sequencing. Libraries were purified using magnetic beads to remove all reaction components. The sizes of the libraries were assessed using an Agilent 2100 bioanalyzer (Agilent Technologies, Netherlands). High-throughput sequencing was performed using 100 bp paired-end reads on the NovaSeq 6000 platform (Illumina Inc., USA). Low-quality DNA reads and adapter sequences were removed using fastp 0.23.1. Clean reads were aligned to the reference genome sequence using bowtie2 2.3.4.3. Duplicate reads were removed using samtools 1.17. Peaks in alignment files were identified using MACS2, and differential binding analysis was performed using the Python conorm package 1.2.0 (https://gitlab.com/georgy.m/conorm) [38]. UCSC Genome Browser session link displaying the uploaded methylated DNA immunoprecipitation sequencing (MeDIP-seq) tracks (https://genome-asia.ucsc.edu/s/JihyeY/GCA_000022165.1).

### 6mA-based DNA digestion assay using DpnII

Fractional 6mA methylation at defined GATC sites was quantified using a quantitative PCR (qPCR)-based assay as previously described [39]. Genomic DNA was extracted using the Wizard^®^ Genomic DNA Purification Kit (Promega) according to the manufacturer’s protocol and eluted in 300 μl of elution buffer. A 35 μl aliquot of gDNA was incubated for 3 h at 37 °C with or without 2 units of DpnII (NEB), followed by heat inactivation at 65 °C for 15 min. To quantify hemimethylated fractions, genomic DNA was split into aliquots and subjected to sequential DpnII→DpnI digestion prior to qPCR. DpnII cleaves only unmethylated GATC, and DpnI cleaves only fully methylated GATC, whereas hemimethylated DNA resists both enzymes. After DpnII digestion and heat inactivation, samples were treated with DpnI (2 U, 37°C, 3 h). Four qPCR reactions were performed for each site (i) digested GATC, (ii) undigested GATC, (iii) digested reference, and (iv) undigested reference. Primer pairs were designed to flank either the GATC site of interest or a non-GATC reference region (Supplementary Table S4). The fraction of methylated DNA was calculated from the resulting Ct values using the ΔΔCt method.

### Materials and methods: marker frequency analyses

For qPCR-based marker frequency analyses (MFA), relative amounts of ori region and terminus region (*dif* gene) DNA were determined by qPCR-based MFA of origin:terminus ratio (*ori*:*ter*) using ΔΔCt with primer efficiencies validated by standard curves [40]. The ratio was determined using the 2−ΔΔCt method (ΔCt = Ct*_ori_* − Ct*_ter_*; ΔΔCt= ΔCt_test_− ΔCt_reference_). The MFA for each sample is the average value of three technical replicates. The reference was genomic DNA from the same bacterial strain grown to stationary phase (overnight culture in LB at 37°C), whose ratio is expected to be 1, is used for the normalization during each run [41]. To corroborate qPCR-based MFA, single-molecule real-time sequencing (SMRT-seq) data were binned into non-overlapping 20 kb windows across the chromosome. For each 20-kb bin, we computed normalized coverage as the number of GATC motif positions covered by primary reads, irrespective of modification status (methylated or unmethylated), divided by the library-wide total.

### Protein purification

To purify IntA and scIHF, the *E. coli* BL21 (DE3) strain containing the pET-28b(+)::*intA* or pET-28b(+)::*scIHF* plasmid was grown in LB medium at 37°C up to an OD_600_ of 0.5 (Supplementary Table S3). To construct pET-28b(+)::*scIHF*, the single-chain product of *ihfA*–linker(GSGGG)–*ihfB–*6 × His was cloned into the pET-28b(+) expression vector, as reported previously [42, 43]. Gene expression was induced by adding 0.5 mM of isopropyl β-d-1-thiogalactopyranoside (IPTG), and the culture was incubated at 37°C for 2 h. Cells were recovered by centrifugation, resuspended in PBS, and disrupted by sonication (10 cycles of 30 s at 40% intensity on ice, with a 30-s pause between each cycle). The lysate was cleared by centrifugation (15 000 × *g*, 4°C, 30 min). IntA–His and scIHF–His were purified using the HisPur^™^ Ni–NTA Purification Kit (Thermo Fisher Scientific, USA). After equilibration with an equilibration buffer (PBS with 10 mM imidazole), the lysate was injected, and unbound proteins were then washed with a washing buffer (PBS with 25 mM imidazole). IntA was eluted with an elution buffer (PBS with 250 mM imidazole). The eluted proteins were monitored by SDS–PAGE, followed by Coomassie staining. After purification, the buffer of purified IntA–His or scIHF–His was exchanged with buffer H [50 mM Tris (pH 8) and 150 mM NaCl] using Amicon^®^ Ultra 3 kDa Centrifugal Filter Units (Merck KGaA, Germany). The resulting samples were aliquoted, snap-frozen in liquid N_2_, and stored at −80°C.

### *In vitro* recombination assay

Reaction mixtures (20 μl) contained 0.08 pmol (300 ng) of a supercoiled plasmid carrying the *attP* site (pSuiAb::*attP*), 0.08 pmol (15 ng) of a 290-bp fluorescent (Cy3)-labeled linear *attB* fragment obtained by the PCR amplification of ligated *attL* and *attR* using the primer pair attL_attB_F(KpnI)/attR_attB_R_Cy3, 7.2 pmol (300 ng) of IntA–His, and 40 μg of boiled BL21 crude extract (95°C, 10 min) in 1× TENDP buffer [25 mM Tris (pH 7.5), 1 mM EDTA, 150 mM NaCl, 1 mM Dithiothreitol (DTT), and 10% PEG8000], as reported previously (Debatisse *et al.*, 2024). The reaction mixtures were incubated at 42°C for 1 h, and the reaction was stopped by adding 0.1% SDS. The samples were analyzed by electrophoresis on 0.8% agarose gels. Fluorescence was assessed using the iBright^™^ FL1500 Imaging System (Invitrogen, USA).

### Electrophoretic mobility shift assay

Promoter regions of *intA* with or without an integration host factor (IHF)-binding site were amplified by PCR and purified using Expin^™^ Cleanup SV (GeneAll, Republic of Korea). Binding reactions (20 μl) were performed with 0.87 pmol of PCR product in a buffer containing 25 mM Tris (pH 8), 75 mM NaCl, 10% glycerol, 0.5 mM DTT, 0.5 mM EDTA, 1 μg poly(dIdC) (Thermo Fisher Scientific, USA), and 0.1 mg/ml BSA. Purified scIHF–His was then added, and the reaction was performed at 37°C for 10 min. The samples were loaded onto nondenaturing 4%–15% polyacrylamide gels (Mini-PROTEAN TGX, Bio-Rad, USA). The gels were run at 4°C at 75 V for 2.5 h and then stained with SYBR^™^ Safe DNA Gel Stain in 0.5× TBE (Thermo Fisher Scientific, USA). Fluorescence was assessed using the iBright^™^ FL1500 Imaging System (Invitrogen, USA).

### Fluorescence-activated cell sorting (FACS)

*S*. Typhimurium WT cells were harvested (5 ml) at each stage of the infection cycle (stages 1–5) and prepared for the live/dead cell staining assay. The cell cultures were adjusted to a final concentration of 5  ×  10^7^ CFU/ml and stained with SYTO9 (1 μg/ml, Thermo Fisher Scientific, USA) and propidium iodide (PI, 1 μg/ml; Invitrogen, USA) for 30 min at RT in the dark. The stained cells were analyzed by assessing fluorescence at excitation wavelengths of 483 and 493 nm and emission wavelengths of 503 and 636 nm for the detection of SYTO9 and PI, respectively, using the Accuri C6 Plus Flow Cytometer (BD Biosciences, USA). The forward scatter threshold was 10 000. Three independent experiments were performed for each condition, with an average of 50 000 cells analyzed per experiment. The proportion of dead/damaged/unstained/live cells was quantified by calculating the percentage of cells stained with SYTO9 following treatment with PI relative to untreated cells. Flow cytometric analyses were conducted in triplicate.

### Quantitative PCR for prophage DNA

To quantify intracellular ST64B, pelleted bacteria were resuspended in 100 μl of water and boiled for 10 min at 95°C [22]. The supernatant was filtered (0.22 μm pore size) and treated twice with phenol–chloroform. DNA was then precipitated with ice-cold isopropanol, as indicated in a previous study [44]. Subsequently, the DNA-containing pellet was resuspended in an appropriate volume of TE buffer (10 mM Tris–HCl, 1 mM EDTA, pH 8.0). qPCR was performed using SYBR^™^ Green Universal Master Mix (Thermo Fisher Scientific, USA) with 20 μl reaction volumes in 96-well plates (Applied Biosystems, USA). The qPCR program was run on the QuantStudio 3 Real-Time PCR System (Thermo Fisher Scientific, USA). qPCR data were extracted from the QuantStudio web application (Thermo Fisher Scientific, USA). Technical duplicates were run for the primer pair targeting the *attP* site of each ST64B. qPCR data were analyzed using the ΔCt or ΔΔCt method, as indicated. Prophage qPCR targets were normalized to the chromosome marker *ydhJ*, which is located distant from the origin and terminus of replication, to minimize confounding effects due to replication bias [45].

### Membrane permeability assays

The ethidium bromide (EtBr) uptake assay was performed at stages 1, 3, and 5 using cell cultures adjusted to an OD_600_ of 0.5, according to a previously described method [32]. Cells were harvested by centrifugation at RT and washed twice with K-phosphate buffer (50 mm, pH 7) by centrifugation at 4000 × *g* for 5 min at RT. The harvested cells were added to the same K-phosphate buffer (5 ml) containing the proton conductor carbonyl cyanide 3-chlorophenylhydrazone (CCCP, 100 μM), which acts as an energy uncoupler and collapses the membrane energy involved in the efflux process [46]. After the addition of EtBr (5 μg/ml), the fluorescence of the EtBr–nucleic acid complex generated by the influx of EtBr into the cells was assessed using a Spark^®^ microplate reader (TECAN, Männedorf, Switzerland) at an excitation wavelength of 515 nm and emission wavelength of 590 nm. For the crystal violet (CV) uptake assay, cells (OD_600_ = 0.5) were harvested by centrifugation at 4000 × *g* for 5 min at RT. The cells were then resuspended in PBS containing CV (10 μg/ml) and incubated for 10 min at 37°C. Subsequently, the stained cells were centrifuged at 15 000 × *g* for 15 min, and the remaining CV without cells was assessed by measuring absorbance at OD_590_. For the 1-N-phenylnaphthylamine (NPN) uptake assay, 100 μl of cells (OD_600_ = 0.5) were resuspended in Hydroxyethyl piperazine Ethane Sulfonicacid (HEPES, 5 mm, pH 7.2) and treated with 100 μl of NPN (20 μM) in a 96-well plate. NPN was added immediately before the measurement of fluorescence. The fluorescence intensity of NPN, produced by the influx of NPN into the cells, was measured using a Spark^®^ microplate reader at an excitation wavelength of 340 nm and emission wavelength of 405 nm. The measurements were performed within 3 min of NPN treatment. All experiments were performed in triplicate.

### Morphological observations and bioinformatic analyses

*S*. Typhurium WT and Δdam cells were prepared for field-emission scanning electron microscopy (FE-SEM) at each infection stage (1–5). Both WT and *Δdam* cells were fixed and prepared according to a previously described method [47]. The cells were observed using SEM (Quanta FEG 250, FEI, USA) at ×40 000 magnification. The length of both WT and *Δdam* cells (*n* = 30 each) was measured using ImageJ software (National Institutes of Health, USA). The promoter regions, transcription factors, and Nucleoid-associated protein (NAP)-binding sites in the upstream region of *intA* were predicted using the web-based tools BPROM (http://www.softberry.com) and P2RP (http://www.p2rp.org), as mentioned previously [48, 49]. The DNA sequences of *intA* with respective codons and the upstream 5′-UTR sequences of *intA* in 17 genomes of bacterial species were directly aligned and visualized using Jalview v. 2.11.3.3 (https://www.jalview.org/). The structures of IntA, IntA–His, the integrase of ST64B, and scIHF–His were predicted using AlphaFold3 (https://alphafoldserver.com). The rank 1 model was selected for visualization using ChimeraX v.1.9 (https://www.cgl.ucsf.edu/chimerax). Synteny analysis was performed using the MaGe software package (https://mage.genoscope.cns.fr/microscope/mage/viewer.php), according to previous studies [33, 50].

### Quantification and statistical analysis

Statistical analyses were performed using GraphPad Prism 9.1.3 (GraphPad Software). Significant differences were determined using Student’s *t*-test (two-tailed) for comparisons between two groups. To evaluate pairwise differences in relative gene expression within each stage, statistical significance was determined using one-way ANOVA followed by Tukey’s multiple comparisons test. The *P-*values are indicated on each figure (*P < 0.05, **P < 0.01, ***P < 0.001, and ****P < 0.0001. ns, not significant).

## Results

### Stage-specific roles of Dam in intracellular stress adaptation

To assess the Dam-mediated epigenetic regulation of *Salmonella* during infection, we established an *in vitro* model mimicking five distinct stages of the *S*. Typhimurium infection cycle (Fig. 1A). These stages represent the transition of the bacterium from the intestinal lumen (stage 1), through SCV formation (stage 2) and maturation (stage 3), to the post-SCV phases (stages 4 and 5). To simulate these environments, we used standard LB medium (pH 7.6, 10 mM Mg^2+^) for stages 1, 4, and 5 and LPM (pH 5.8, 8 μM Mg^2+^) for stages 2 and 3, following established protocols. We modeled oxidative stress encountered during early SCV formation by supplementing the stage 2 medium with 2 mM H_2_O_2_ [8, 51]. To mimic the conditions of late-stage SCV maturation and effector secretion, we incubated the cells in LPM for 20 h [52]. To further validate the physiological relevance of our *in vitro* infection cycle model, we assessed the expression of *sipA* and *ssaC*, representative virulence genes from SPI-1 and SPI-2, respectively (Supplementary Fig. S1A) [28, 29]. In agreement with the established *in vivo* infection program, *sipA* expression declined at stages 2 and 3, while *ssaC* expression increased significantly over time (Supplementary Fig. S1B). These trends were corroborated by promoter activity measurements using sfGFP transcriptional reporters, which exhibited reciprocal activation patterns between stages 1 and 3 (Supplementary Fig. S1C). Together, these findings confirm that our *in vitro* system recapitulates key regulatory transitions associated with virulence gene expression within the SCV. During the infection cycle, *dam* expression initially declined but peaked ∼60 min after the medium shift (stage 2), reaching levels >20-fold higher than those observed at stage 1 (Fig. 1B and Supplementary Fig. S2A) [8]. This biphasic expression pattern is consistent with previous reports of transient *dam* repression followed by gradual upregulation under oxidative stress. These findings suggest that *dam* is acutely repressed during the early stress response but becomes strongly induced during the later adaptation phase. However, *dam* transcript levels decreased (∼1.4-fold) by stage 3, indicating that the extreme upregulation was transient (Fig. 1B, and Supplementary Fig. S2A and B). The estimated half-life of *dam* mRNA under these conditions was 2.2 min, suggesting rapid degradation following transcriptional downregulation (Fig. 1C). Interestingly, although oxidative stress (H_2_O_2_) was not required for the transcriptional induction of *dam* at stage 2, it significantly impaired translation, resulting in minimal accumulation of the Dam–msfGFP fusion protein at this stage (Fig. 1C and D, and Supplementary Fig. S1). Notably, we detected increased Dam–msfGFP levels at stage 3, potentially reflecting the translation of mRNAs transcribed earlier during stage 2. This lag may result from H_2_O_2_-induced effects on protein folding or translational repression [53]. Furthermore, elevated Dam–msfGFP levels persisted into stages 4 and 5, even after the removal of SCV-associated stressors; however, the mechanisms underlying this prolonged expression remain unknown. Although the Dam–msfGFP reporter system does not enable the precise quantification of endogenous Dam protein, our results suggest that Dam-mediated methylation activity may remain elevated throughout later stages of infection.

During the SCV phase, the WT 14028s strain exhibited characteristic biphasic growth. However, the *Δdam* mutant exhibited significant growth impairment specifically under these conditions, underscoring the importance of Dam-dependent methylation in adaptation to SCV-associated stress (Fig. 1E). Interestingly, WT cells gradually adopted an elongated morphology during SCV stages. Heterogeneous cell shapes emerged at later time points, with both elongated (2.5–3.5 μm) and short (0.5–0.7 μm) forms observed at stages 4 and 5 (Supplementary Fig. S3A and B). In contrast, the *Δdam* mutant exhibited cell elongation as early as stage 1, suggesting a defect in cell division even before SCV stress (Supplementary Fig. S3A and C). As morphological changes are frequently associated with bacterial stress responses, particularly alterations in membrane integrity and viability, we assessed live/dead cell ratios throughout the infection cycle in WT populations [54, 55]. Although SYTO9 staining remained relatively stable across stages, PI fluorescence began increasing at stage 2 and peaked at stage 4 (75.5%) (Supplementary Fig. S3D). Importantly, increased PI uptake does not necessarily indicate cell death. Instead, it reflects disrupted membrane integrity (Fig. 1E and Supplementary Fig. S3D) [56, 57]. These morphological changes coincided with altered membrane permeability. Under SCV-mimicking conditions, WT cells exhibited 25%–40% higher NPN fluorescence and 7.1-fold higher CV retention than WT cells in LB medium (stages 1 and 5), indicating enhanced membrane permeability (Supplementary Fig. S3E and F). Moreover, EtBr accumulation increased by 10.3% at stage 3 in WT cells, consistent with increased phospholipid-mediated influx under SCV stress (Supplementary Fig. S3G). Notably, *Δdam* cells failed to exhibit these adaptive membrane responses. Instead, they maintained constitutively elevated levels of NPN uptake (∼1.2-fold higher than WT cells), CV retention (∼7.2-fold higher), and EtBr accumulation (∼1.2-fold higher) at all stages (Supplementary Fig. S3E–G). Collectively, these results highlight the key role of Dam-mediated methylation in preserving membrane integrity and regulating growth during SCV-associated stress.

Although the epigenetic control of bacterial morphology, such as cell elongation and membrane dynamics, has been well characterized in several bacterial species such as *E. coli* and *Acinetobacter baumannii*, how such regulation influences prophage behavior remains unclear [34, 58]. Notably, a previous study has reported enhanced excision of the cryptic prophage ST64B in *S*. Typhimurium LT2 strains that lack Dam; however, the molecular basis underlying this phenomenon remains still elusive [44]. To further assess this regulatory mechanism, we examined prophage dynamics using our staged infection cycle model. We quantified the excision of ST64B by measuring the *attP* site, which only can detect the circularized phage DNA but not the prophage DNA, normalized to the *ydhJ* locus (a chromosomal region located distant from the origin and terminus of replication) to minimize confounding effects due to replication bias [45]. In WT cells, ST64B excision peaked at stage 2 (SCV formation) and declined at subsequent stages, reflecting a tightly controlled process (Fig. 1F). In contrast, the *Δdam* mutant exhibited persistently high levels of excision across all stages, indicating that Dam-mediated methylation plays a central role in maintaining prophage stability throughout the infection cycle (Fig. 1F). Consistent with previous reports linking SCV-associated stress to ST64B mobilization and increased DNA release in *dam*-deficient strains, we observed substantial accumulation of prophage DNA in the supernatants of WT cell cultures during stages 2 and 3 and in those of *Δdam* cell cultures across all stages (Fig. 1F) [22, 44]. This pattern paralleled the elevated membrane permeability detected in *Δdam* mutants in multiple independent assays (Supplementary Fig. S3E–G), suggesting a link between membrane stress and prophage mobilization. To directly assess the role of Dam in regulating prophage excision, we used a CRISPRi system based on chromosomally integrated dCas9 under the control of an aTc-inducible promoter (Supplementary Fig. S4). Dose-dependent induction of dCas9 to knockdown *dam* expression increased ST64B excision, with a significant increase observed at 50 nM aTc (*P* < 0.0001) and maximal excision levels noted at 100 nM aTc (*P* < 0.0001), comparable to those observed in the *Δdam* strain (Fig. 1G). Collectively, these findings indicate that Dam regulates ST64B perturbation. The activity of Dam enables precise temporal control over ST64B excision, facilitating its induction during SCV-associated stress and repression during post-SCV conditions. This finding highlights a previously unrecognized epigenetic control that links environmental factors to prophage regulation during infection.

### Temporal dynamics of methylation pattern

**Figure 2.**
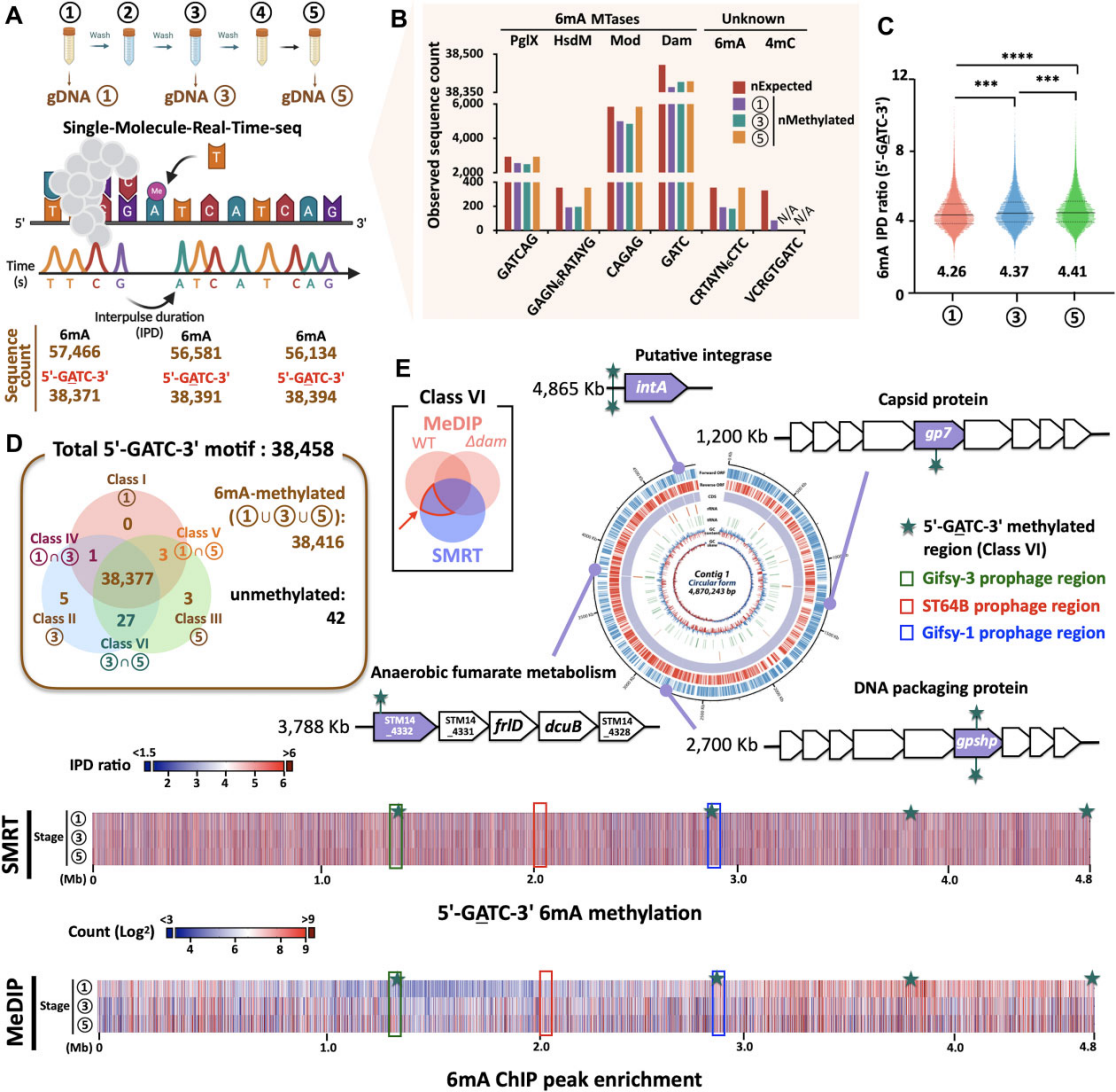
Temporal methylome dynamics during infection. (**A**) SMRT-seq during different infection stages (1, 3, and 5). 6mA and GATC motifs were sorted according to the IPD ratio (>1.5). (**B**) Observed sequence count of expected sequences (nExpected) and methylated sequences (nMethylated) at stages 1, 3, and 5. (**C**) Average 6mA IPD ratio at stages 1, 3, and 5. Each dot point on the violin plot represents a single motif detected at each stage. The *P*-values are indicated on each figure (***P < 0.001 and ****P < 0.0001). (**D**) GATC motif classified according to the methylation status during different stages (Supplementary Table S2). (**E**) Genes related to Class VI type motif refined by MeDIP-seq; the genes were present in the WT strain and absent in the *Δdam* strain (above, Table 1). Stage-specific 6mA methylation patterns were clustered across the 14028s chromosome (below). Heatmaps of methylation patterns were visualized using GraphPad Prism 9.1.3 (GraphPad Software).

To assess the temporal dynamics of DNA methylation during infection, we performed SMRT-seq at stages 1, 3, and 5 of the *Salmonella* infection cycle. Genome-wide mapping revealed widespread methylation of GATC motifs, with over 99.7% of 5′-GATC-3′ sites methylated across all stages (38371–38394 out of 38 458 sites; Fig. 2A and B and Supplementary Table S1). In addition to canonical Dam targets, we identified several 6mA motifs, including the Type I MTase PglX motif (GATCAG), the Type III Mod motif (CAGAG), and a novel palindromic sequence (CRTAYNNNNNNCTC), all of which exhibited the highest methylated sequence count at stage 5 compared to stages 1 and 3 (Fig. 2B and Supplementary Table S1). Notably, a 4mC motif (VCRGTGATC) was exclusively methylated at stage 1 and undetectable at stages 3 and 5, suggesting that this modification is lost during SCV-associated stress and not restored during recovery (Fig. 2B and Supplementary Table S1). Methylation intensity, inferred from mean IPD ratios, progressively increased from stage 1 (4.26) to stage 3 (4.37) and stage 5 (4.41) (*P* < 0.001), indicating the global enhancement of methylation as infection progresses (Fig. 2C). We next classified methylation motifs into six categories based on their stage-specific methylation patterns. Among these, Class VI sites (methylated at stages 3 and 5 but unmethylated at stage 1) were the most prevalent (*n* = 27; Fig. 2D and Supplementary Table S2). These sites were predominantly located in intergenic regions (21 out of 27; 77.8%), indicating potential roles in transcriptional regulation (Supplementary Table S2). Importantly, Class VI loci exhibit persistent methylation at later stages (3–5) despite ongoing cell division (Fig. 1E). This pattern suggests continuous Dam activity on newly replicated DNA and underscores a potential mechanism for epigenetic inheritance in bacteria.

To better understand the dynamics of 6mA modifications, we complemented our SMRT-seq analysis, which detects base modifications via polymerase kinetic signatures, with MeDIP-seq, which enriches for 6mA-modified DNA independently of sequence context (Supplementary Fig. S5A) [37]. Chromosomal profiling of MeDIP peaks in WT cells revealed a transition from discrete, localized enrichment at stage 1 to a more broadly distributed methylation pattern by stage 3; this pattern was sustained and intensified at stage 5 (Supplementary Fig. S5B). These findings suggest the progressive amplification and maintenance of methylation marks initially established during SCV-associated stress. In contrast, *Δdam* mutants showed uniformly low 6mA signals across all stages, exhibiting little variation (Supplementary Fig. S5B). Metagene analyses of 6mA distribution around transcription start sites (TSSs) further revealed consistent enrichment in WT cells, particularly at stages 3 and 5; however, *Δdam* cells exhibited minimal or no enrichment in these regions (Supplementary Fig. S5C). These results support a central role of Dam in establishing stage-specific methylation patterns at transcriptional regulatory sites. Despite the observed mobilization of the ST64B prophage during stage 3, integrated SMRT-seq and MeDIP-seq analyses revealed that differentially methylated regions were not located within the ST64B genome itself but were instead mapped to four Class VI loci elsewhere on the chromosome (Fig. 2E and Table 1). Among these, a regulatory region upstream of *intA*, which encodes a putative tyrosine-type prophage integrase, exhibited significant hypermethylation in WT cells at both stage 3 (IPD ratios: 4.35 and 3.78) and stage 5 (IPD ratios: 5.32 and 4.42) (Fig. 2E and Table 1). Although *intA* shares only 50.2% sequence identity with the canonical ST64B integrase *int* (which is encoded in the prophage genome), the temporal correlation between *intA* hypermethylation and the subsequent reduction of excised ST64B prophage levels (stages 3–5) in WT cells led us to hypothesize that methylation at the *intA* regulatory region modulates integrase expression and mediates the reintegration of ST64B.

**Table 1.** Genes related to Class VI methylated loci. Genomic loci exhibiting stage-specific methylation patterns (Class VI: methylated at stages 3 and 5) identified via SMRT/MeDIP-seq.

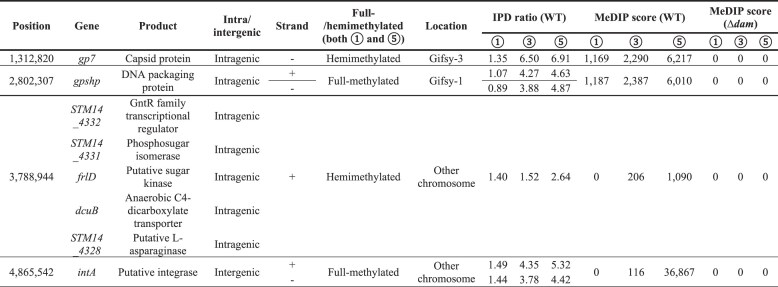

**Figure 3.**
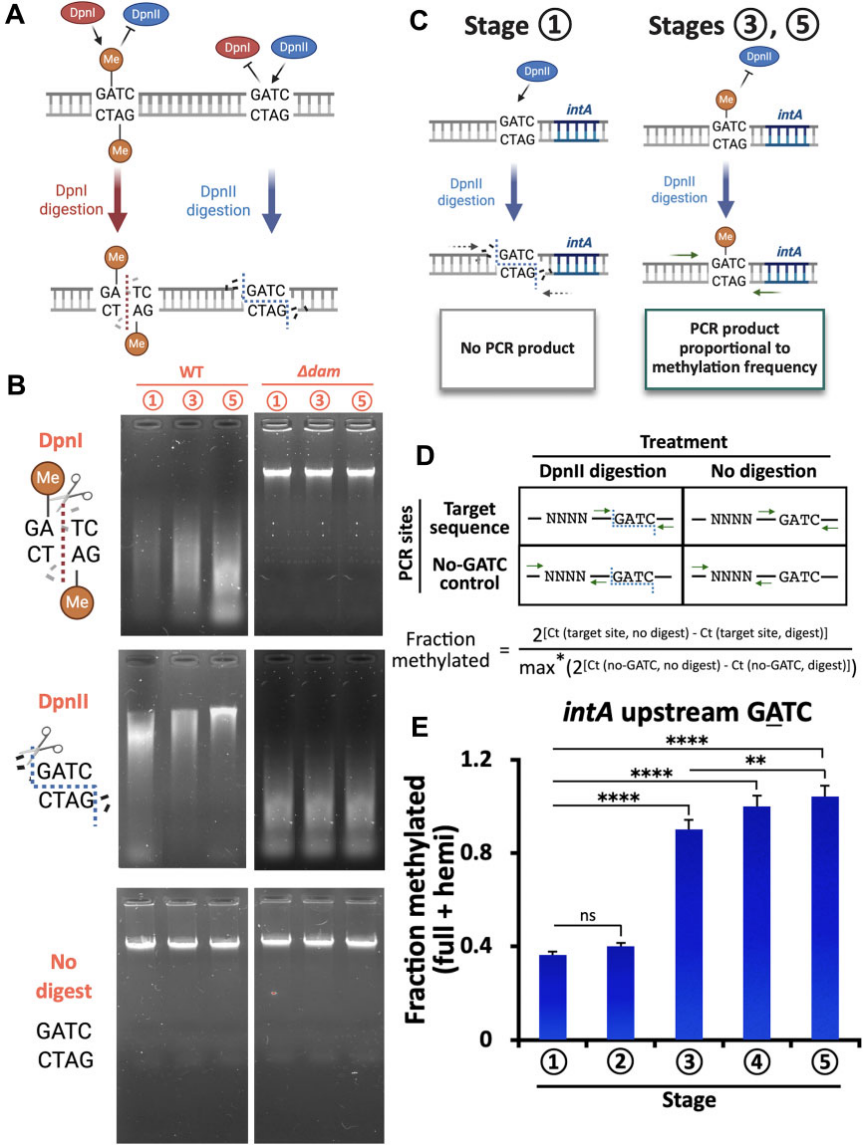
Establishment of a quantitative 6mA assay to measure methylation frequency at GATC sites. (**A**) Schematic illustrating selective digestion by DpnI (which cuts only methylated GATC) and DpnII (which cuts only unmethylated GATC). (**B**) Agarose gel electrophoresis confirming selective digestion patterns using genomic DNA from WT and *Δdam* strains at stages 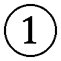, 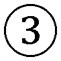, and 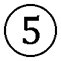. (**C**) Schematic of qPCR-based methylation assay at the *intA* promoter. DpnII digestion prevents amplification of unmethylated DNA, while methylated templates remain amplifiable. (**D**) Primer design and method of calculating methylation fraction based on *C*_t_ values. *max, the maximum value of all calculated fractions. (**E**) Quantification of methylation fraction at the *intA* upstream GATC site across stages 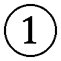
–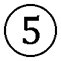 using DpnII-qPCR. Bars represent mean ± SD (*n* = 3); **P < 0.01, ****P < 0.0001 (one-way ANOVA with Tukey’s test).

We next employed a 6mA-sensitive DNA digestion assay using DpnII, to quantitatively assess methylation at *intA* regulatory locus (Fig. 3A–D) [39]. Consistent with our SMRT- and MeDIP-seq results, this site-specific 6mA–qPCR assay confirmed a marked increase in methylation at the *intA* upstream GATC site beginning at stage 3 (Fig. 3E). To test whether replication dynamics affect the methylation pattern and the consequent intA expression, we quantified hemimethylated GATC states using sequential DpnII→DpnI qPCR (Supplementary Fig. S6A–C) and directly measured replication by *ori:ter* qPCR and SMRT-seq-based mini-MFA [40]. The *ori:ter* ratio decreased from stage 1 to 2 and remained low through stages 3–5, consistent with mini-MFA showing an *ori*→*ter* gradient at stage 1 (≈ 2.6) but flat profiles at stages 3 and 5 (≈ 1.1) (Supplementary Figs S6D and E, and S7). At an *ori*-proximal GATC, total methylation remained high, whereas hemimethylation declined after stage 1 and paralleled *ori:ter* trends (Supplementary Fig. S6F and G). In contrast, the *intA* upstream GATC showed rising hemimethylation at stages 3–5 (0.20, 0.23, and 0.25) despite low replication (Supplementary Fig. S6H). These findings indicate that the sharp increase in methylation at *intA* during stage 3 cannot be explained by a recovery of replication activity. Instead, the onset of methylation at this locus is more likely due to changes in local Dam accessibility that occur during our infection cycle. To evaluate whether H_2_O_2_ alone induces genome-wide methylation changes, 6mA MeDIP-seq was performed at stage 2. Although the overall methylation profile at this stage remained broadly similar to that of stage 1, a subset of MeDIP peaks showed partial redistribution (Supplementary Fig. S8A and B). These results suggest that oxidative stress may influence methylation at specific loci but does not trigger widespread methylome remodeling. Notably, prominent methylation changes in the upstream region of *intA* emerged predominantly at stage 3 (Fig. 3E). This pattern, together with the observed increase in Dam–msfGFP expression at stage 3 (Fig. 1D), supports the conclusion that the major shift in methylation is primarily driven by the recovery of Dam activity rather than H_2_O_2_ itself.

### Persistence of SCV-induced methylation under post-SCV conditions

**Figure 4.**
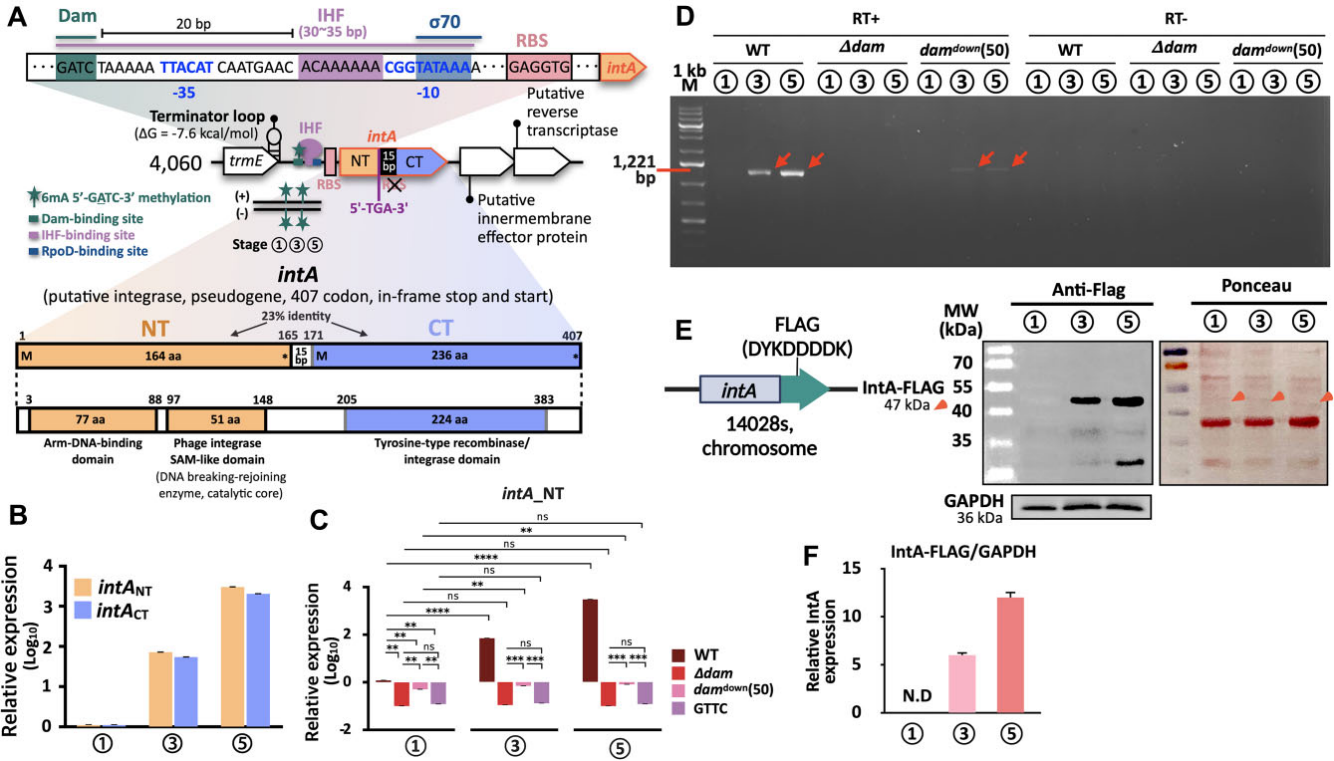
Promoter architecture and expression of *intA*. (**A**) *intA* regulatory region. Predicted −10/−35 boxes are indicated in blue bold characters. The domain structure of *intA* is indicated below. (**B**) Expression of *intA* verified by qRT-PCR using primers detecting each N-terminal/C-terminal domain. (**C**) *intA*_NT_ expression in the *dam-*deficient mutant (Δdam), mutant with *dam* knockdown using 50 nM of aTc [*dam*^down^(50)], and upstream A–T (GTTC) mutant. (**D**) Reverse transcription of *intA* using primers detecting the whole sequence, with (RT+) or without (RT−) the reverse transcriptase of WT and *dam* mutant strains. (**E**) Western blot analysis of IntA–FLAG (47 kDa) detected at stages 3–5 and Ponceau S staining of membrane with transferred crude extract, confirming readthrough of the TGA codon. Experiments were performed three independent times. (**F**) IntA–FLAG expression normalized by GAPDH. Densitometric analysis was performed using ImageJ software (National Institutes of Health, USA).

To explore the regulatory architecture underlying *intA* expression, we assessed SCV-induced methylation at the *intA* promoter beyond the stress phase. Our SMRT-seq analysis revealed that Dam-mediated methylation marks established during SCV stress (stage 3) remained detectable in the post-SCV environment (stage 5). Fine-scale mapping of the *intA* promoter uncovered a GATC motif (the Dam recognition site) located near the −35 box and an IHF-binding motif (5′-ACAAAAAA-3′) positioned ∼20 bp downstream (Fig. 4A). Despite being spatially separated, the extended IHF-binding footprint (∼30–35 nt) creates potential for competitive binding between Dam and IHF at this locus [59, 60]. This organization suggests a regulatory mechanism in which methylation and IHF occupancy are mutually exclusive and modulate *intA* transcription. *intA* encodes a putative 407-aa protein consisting of three domains: an N-terminal DNA-binding domain (77 aa), a phage integrase SAM-like domain (51 aa), and a C-terminal tyrosine recombinase domain (178 aa) (Fig. 4B). Intriguingly, *intA* contains an in-frame stop codon (TGA, internal DNA sequence), followed by a start codon (ATG), characteristic of pseudogene-like architecture. We identified a single ribosome-binding site (5′-GAGGTG-3′) upstream of the main start codon but not within the 15 bp spacer separating the stop and downstream start codons, indicating that translation may be restricted to the primary *intA* transcript (Fig. 4A and B). Building on prior findings that IHF–DNA interactions are influenced by Mg^2+^ availability and growth phase, we hypothesized that IHF dissociates from the *intA* promoter under SCV conditions (stage 3), enabling Dam to methylate the GATC motif [61]. This methylation can sterically inhibit IHF re-binding, thereby maintaining *intA* transcription in the post-SCV phase.

To assess *intA* expression across infection stages, we performed domain-specific qRT-PCR targeting both N-terminal and C-terminal regions relative to the internal stop codon. Despite the presence of a premature stop codon, both domains were transcriptionally induced at stage 3 (∼10^2^-fold) and markedly upregulated at stage 5 (∼10^3^-fold) compared to stage 1 (Fig. 4B). Notably, *intA* transcription was undetectable in both *Δdam* and CRISPRi-mediated *dam*-silenced strains at all stages (Fig. 4C and D). Moreover, site-directed mutation of the GATC methylation motif for disrupting Dam binding entirely abolished *intA* expression, confirming the requirement of Dam methylation for transcriptional activation (Fig. 4C). Consistent with transcriptional data, western blot analysis of the chromosomally integrated *IntA*–FLAG fusion protein revealed stage-specific protein accumulation in WT cells, with initial detection at stage 3 (6.2-fold increase) and further elevation at stage 5 (12.4-fold increase) (Fig. 4E and F). Remarkably, the FLAG antibody detected the C-terminal epitope despite the internal TGA stop codon, indicating translational readthrough and production of the full-length integrase protein. The observed molecular weight (47 kDa) matched the predicted size of the complete *IntA*–FLAG fusion (428 aa; 406-aa IntA with 22-aa FLAG tag) (Fig. 4F). Considering prior evidence of bacterial codon readthrough, particularly at TGA, these results suggest that context-dependent suppression of the internal stop codon enables full-length *IntA* translation during infection [62, 63]. Together, these findings indicate that Dam-dependent methylation of the *intA* promoter is a crucial epigenetic switch controlling integrase production, with functional consequences mediated through stress-induced stop codon readthrough during the infection cycle.

### Reintegration of ST64B mediated by IntA expression

**Figure 5.**
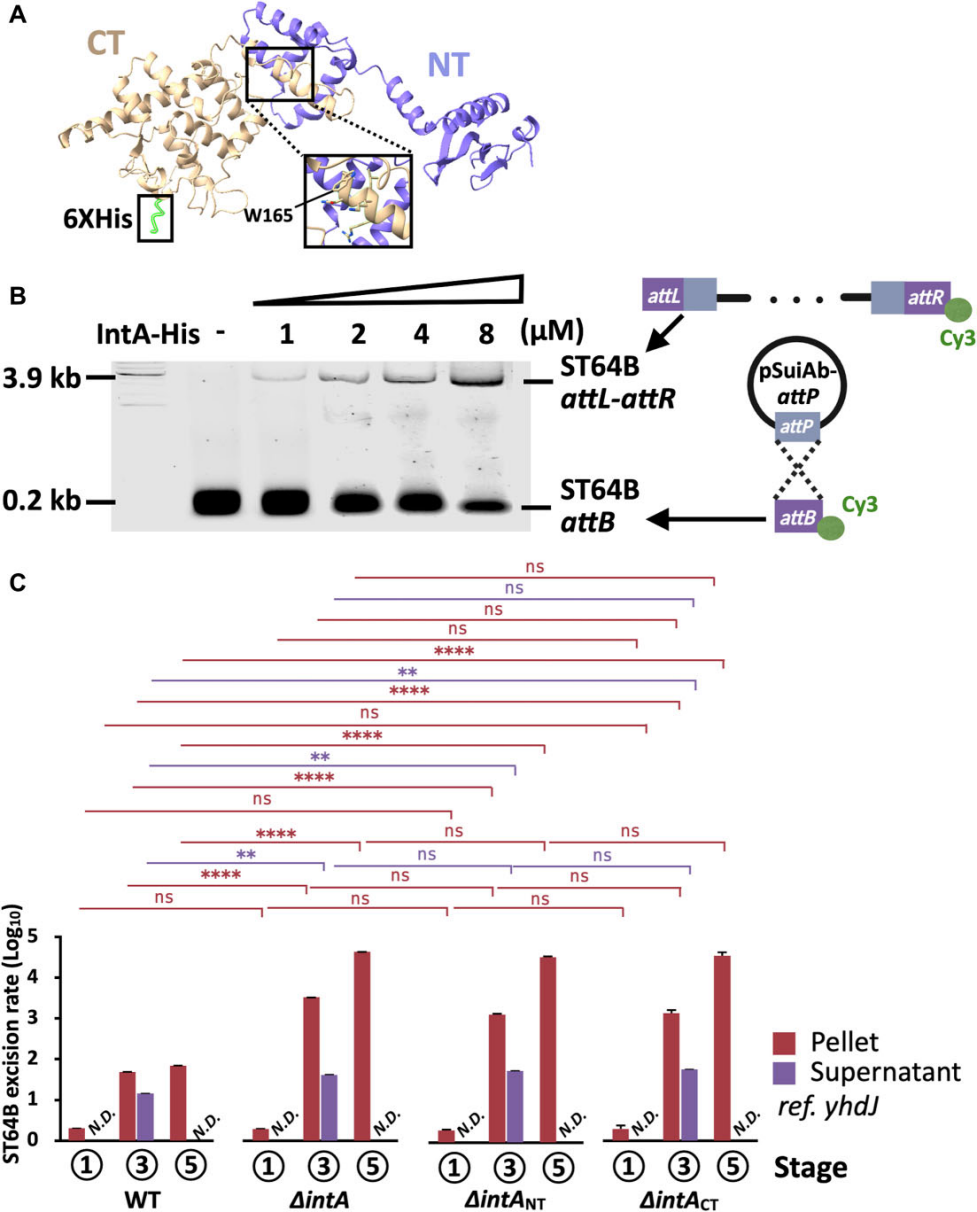
IntA-mediated ST64B reintegration. (**A**) Homology model of IntA–His constructed using AlphaFold3 visualized with ChimeraX. The internal stop codon was considered as tryptophan (W165), according to previous studies (Romero Romero *et al.*, 2024; Toledano *et al.*, 2024). (**B**) *In vitro* recombination assay. IntA–His (1–8 μM) integrates the pSuiAb–*attP* plasmid into linear *attB–*Cy3, yielding a 3.9 kb product. (**C**) ST64B excision rates of WT and region-specific deletion mutants (*ΔintA*_NT_ and *ΔintA*_CT_). qPCR of *attP* normalized by *ydhJ*. All experiments were performed in triplicate. Statistical comparisons were performed using one-way ANOVA with Tukey’s multiple comparison test, among stages within each strain and between strains at the same stage, with significance levels indicated in the figures (**P < 0.01 and ****P < 0.0001. ns, not significant).

As *intA* expression is regulated by Dam-dependent methylation, we next assessed whether IntA directly mediates ST64B recombination at the bacterial *att* site. Based on prior reports of stop codon readthrough, we assumed that the internal TGA codon within *intA* is decoded as tryptophan (TGG codon) during translation (Fig. 5A) [62, 63]. We confirmed the expression of C-terminal His-tagged IntA in *E. coli* BL21, with monocistronic transcription verified by qRT-PCR (Supplementary Fig. S9A and B). The truncated N-terminal product (∼19 kDa) was predominant in whole-cell lysates, while full-length IntA–His (∼45 kDa) was recovered by Ni–NTA affinity purification, indicating translational readthrough of the internal stop codon (Supplementary Fig. S9C and D). We subjected purified IntA–His to *in vitro* recombination assays using supercoiled *attP*-containing DNA and 3′-Cy3-labeled linear DNA harboring the *attB* site [63]. Reactions containing 1–8 μM IntA resulted in the concentration-dependent formation of a 3.9 kb *attL–attR* recombination product, with a corresponding decrease in the *attB* substrate (Fig. 5B). Remarkably, IntA catalyzed the integration reaction in the absence of canonical cofactors, such as IHF or Fis, similar to the host-independent tyrosine integrase ^mv4^Int of *Lactobacillus delbrueckii* [64]. These findings establish IntA as a *bona fide* integrase that can mediate ST64B recombination alongside the prophage-encoded canonical integrase. To assess the *in vivo* function of IntA during infection, we constructed three targeted *intA* mutants: a complete deletion mutation (Δ*intA*) and two domain-specific deletion mutants targeting the N-terminal DNA-binding/phage integrase domain (Δ*intA*_NT_, aa 1–165) and the C-terminal tyrosine-type recombinase domain (Δ*intA*_CT_, aa 171–407), corresponding to regions upstream and downstream of the internal stop codon, respectively (Figs 4A and 5C). We quantified ST64B excision at stages 1, 3, and 5 by performing qPCR using both cell pellet and supernatant fractions. At stages 3 and 5, all tested mutants exhibited markedly higher excision than the WT strain, with log_10_ values reaching 3.5–4.6 in cellular DNA (Fig. 5C). In the supernatant, we exclusively detected ST64B DNA at stage 3, with higher levels noted in knockout strains (log_10_ ∼1.5) than in the WT strain. These trends were inversely correlated with *intA* mRNA and IntA protein expression levels in WT cells, both of which peaked during stages 3–5 (Figs 4C–G and 5C), supporting a role of IntA in prophage reintegration during the late stages of infection. Notably, domain-specific mutants (Δ*intA*_NT_ and Δ*intA*_CT_) phenocopied the full *intA* deletion, indicating that both N-terminal and C-terminal domains are essential for IntA function (Fig. 5C). These results suggest that full-length IntA, produced by stop codon readthrough, is necessary for the coordinated control of ST64B stability, particularly during SCV-associated stress and post-SCV recovery.

### Sustained *intA* expression mediated by IHF–Dam promoter switch

**Figure 6.**
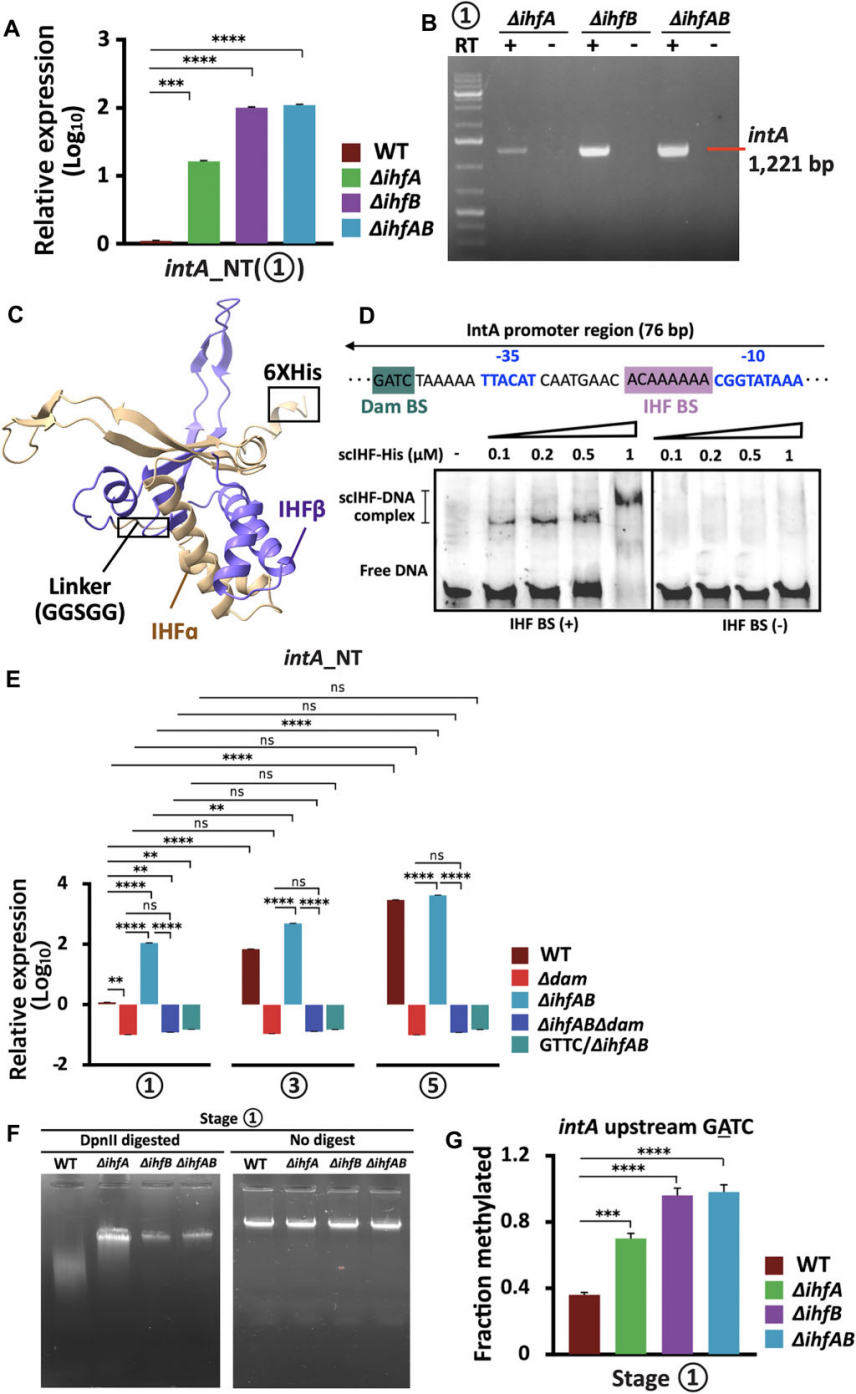
IHF–Dam epigenetic switch. (**A**) Relative expression of *intA*_NT_ at stage 1 in WT and IHF mutants. (**B**) Reverse transcription of the *intA* whole sequence with (RT+) or without (RT−) the reverse transcriptase of IHF mutant strains at stage 1. (**C**) Homology model of scIHF–His constructed using AlphaFold3 visualized with ChimeraX. The alpha subunit (IHFα, beige) and beta subunit (IHFβ, purple) are linked with the GGSGG linker. (**D**) EMSA of scIHF–His and the *intA* promoter with the IHF-binding site (IHF BS+/−). (**E**) Relative *intA*_NT_ expression at stages 1, 3, and 5 in the WT strain and Δdam, ΔihfAB, and upstream *intA* A–T mutants. (**F**) Agarose gel electrophoresis after DpnII digestion of genomic DNA from WT and *Δihf* mutants at stage 1. (**G**) Quantification of methylation levels at the *intA* upstream GATC using qPCR following DpnII digestion. The P-values are indicated on each figure (**P < 0.01, ***P < 0.001, and ****P < 0.0001. ns, not significant).

Our *in silico* analysis identified a putative IHF-binding motif located ∼20 bp downstream of a Dam-recognized GATC site in the *intA* promoter region (Fig. 4A), suggesting competitive interference between Dam and IHF for promoter occupancy. To determine whether IHF functions as a repressor or an activator of *intA*, we analyzed *intA* expression in IHF-deficient strains lacking the α subunit (Δ*ihfA*), the β subunit (Δ*ihfB*), or both (Δ*ihfAB*). All mutants exhibited significantly increased expression of the N-terminal domain of *intA* (*intA*_NT_) at stage 1, with the Δ*ihfA* mutant showing a 1.2 log_10_-fold increase and both Δ*ihfB* and Δ*ihfAB* mutants showing ∼2.0 log_10_-fold increases relative to the WT strain (Fig. 6A). These findings indicate that IHF, through either subunit, acts as a strong transcriptional repressor of *intA* under non-stress conditions. RT-PCR further confirmed robust expression of *intA* transcripts in all IHF-deficient strains at stage 1; however, we did not detect any *intA* transcript in the WT strain under the same conditions (Figs 4C and D, and 6A and B). To directly assess IHF binding, we performed electrophoretic mobility shift assays (EMSAs) using a purified single-chain IHF–His protein (scIHF–His) and DNA probes spanning the *intA* promoter (Fig. 6C). scIHF–His specifically bound to the WT probe containing the IHF-binding site [IHF BS(+)] in a concentration-dependent manner (0.1–1 μM); however, we did not observe any binding with the probe lacking this site [IHF BS(−)] (Fig. 6D). At 1 μM IHF, the prominent upward shift in the DNA–protein complex was consistent with severe DNA bending (∼120°), a known outcome of IHF binding rather than multimerization, as confirmed by the absence of shifts in the IHF BS(−) control [65]. The IHF-binding motif partially overlapped with the predicted σ^70^ with the –10 promoter element (TATAAAA), implicating IHF in transcriptional repression through the steric hindrance of RNA polymerase recruitment (Fig. 4A) [66]. These findings support a model in which IHF binding at stage 1 blocks *intA* transcription, while stress-induced IHF dissociation during SCV entry allows Dam access to the GATC site, enabling transcriptional activation.

To further dissect this interplay, we compared *intA* expression in the Δ*ihfAB* mutant, the Δ*dam* mutant, and the triple mutant Δ*ihfABΔdam* at stages 1, 3, and 5. The Δ*ihfAB* mutant exhibited increased expression across all stages (up to ∼4 log_10_-fold increase at stage 5); however, *intA* expression was abolished in the Δ*ihfABΔdam* mutant (Fig. 6E). Similarly, GATC promoter mutation (GTTC) in the Δ*ihfAB* background suppressed *intA* expression, indicating that Dam-mediated methylation is essential for promoter activation even in the absence of IHF repression (Fig. 6E). In WT cells, the activation of *intA* at stages 3 and 5 coincided with GATC methylation at the promoter, supporting the following sequential regulatory mechanism: (i) IHF dissociation under SCV stress, (ii) Dam methylation of the exposed GATC motif, and (iii) methylation-dependent inhibition of IHF re-binding in the post-SCV environment (Fig. 6E and Table 1). The opposing expression profiles of Δ*ihfAB* relative to Δ*ihfABΔdam* and GTTC/Δ*ihfAB* strains across all stages underscore the requirement of Dam activity for maintaining *intA* expression after SCV stress resolution (Fig. 6E). To directly assess whether IHF restricts Dam-dependent methylation, we quantified methylation frequency at the *intA* upstream GATC site in Δ*ihfA*, Δ*ihfB*, and Δ*ihfAB* mutants using the DpnII-qPCR assay during stage 1. All Δ*ihf* strains exhibited significantly elevated methylation levels compared to WT across the chromosome, including at the *intA* upstream region, indicating that IHF binding interferes with Dam access and permits the maintenance of partially methylated states (Fig. 6F and G).

**Figure 7.**
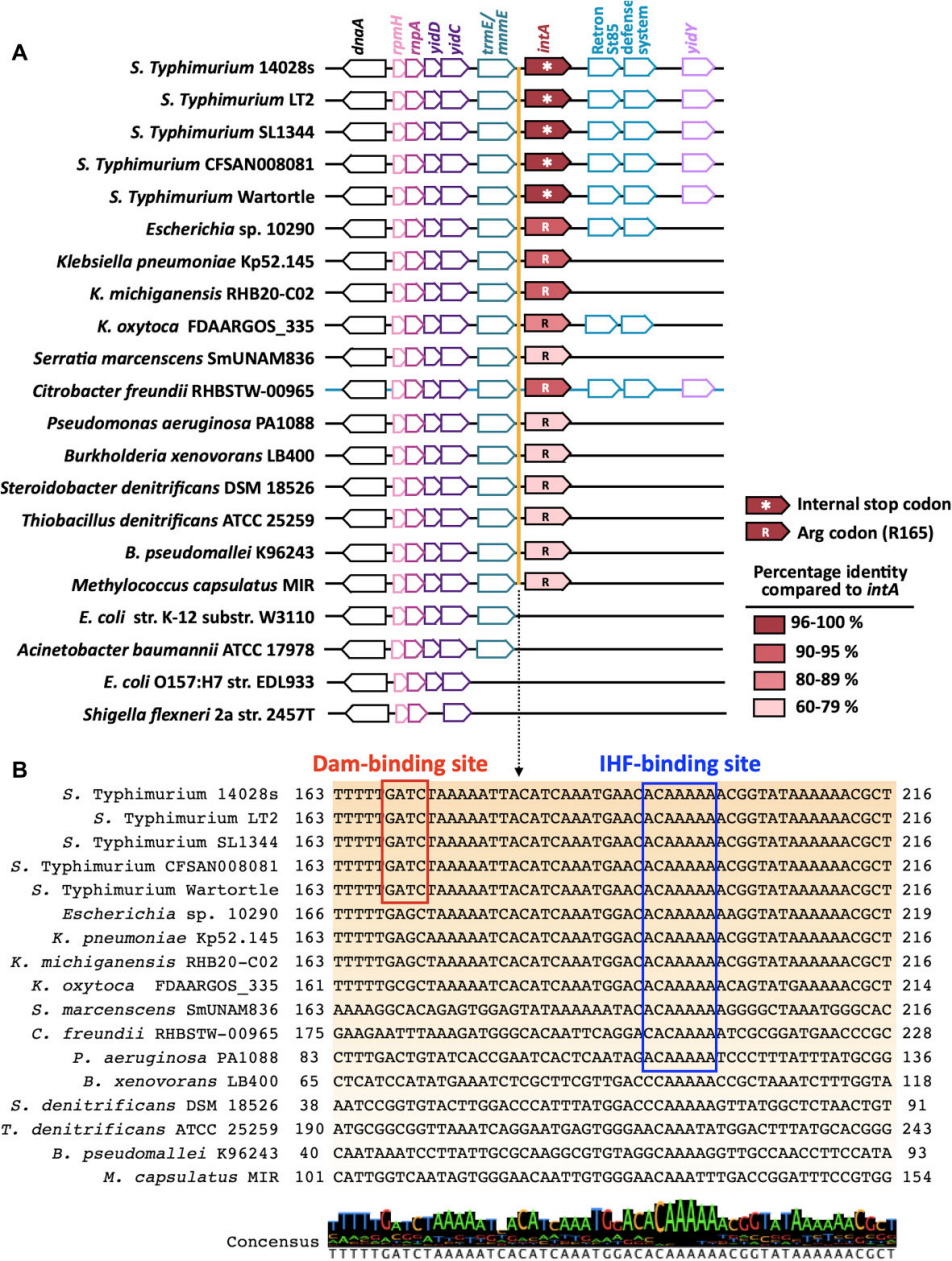
IntA synteny and conservation of promoter Dam/IHF-binding sites across various bacterial species. (**A**) Synteny cluster of *intA* across 21 species. The *intA* alleles contain internal stop codon (*) or arginine (R) codon substitutions. Synteny analysis was performed using the MaGe software package. (**B**) Promoter motif conservation of Dam- and IHF-binding sites. DNA sequences of the upstream 5′-UTR of *intA* in 17 genomes of the tested bacterial species were directly aligned and visualized using Jalview v. 2.11.3.3.

Comparative genomic analysis revealed conserved *intA* synteny in 17 out of 21 surveyed bacterial species, typically located downstream of *trmE*, within a putative defense island enriched for viral elements and retron-associated genes (Fig. 7A) [67, 68]. Notably, *intA* alleles displayed evolutionary divergence, with internal stop codons (TGA) or arginine substitutions [R165, CGA(C)] identified in distinct species, suggesting independent horizontal acquisition events or adaptive remodeling under host-selective pressures (Fig. 7A). Promoter analysis further showed that the Dam-binding motif is primarily conserved within *Salmonella* species, whereas the IHF-binding motif is more broadly conserved across diverse genera, including *Klebsiella*, *Serratia*, and *Citrobacter* (Fig. 7B).

## Discussion

The intracellular lifestyle of *S. enterica* requires dynamic regulatory mechanisms to adapt to the hostile environment of the SCV, which is characterized by nutrient deprivation, acidic pH, limited Mg^2+^, and exposure to ROS (Fig. 1A). Upon transition into the SCV, elevated levels of H_2_O_2_ disrupt *de novo* protein synthesis, likely through transfer RNA (tRNA) fragmentation, as reported previously [22, 53]. The observed decrease in Dam protein levels at stage 2 reflects accelerated RNA decay and translational inhibition under oxidative stress (Fig. 1C and D). The resulting Dam deficiency leads to marked cell elongation and increased membrane permeability, recapitulating the phenotypes observed in Δ*dam* mutants (Supplementary Fig. S3). Although our *in vitro* system cannot fully represent the *bona fide* SCV environment, particularly in terms of *in vivo* cell division, the emergence of both elongated and short cell populations during the post-SCV stages may reflect Dam-dependent regulation of cell cycle progression, warranting further investigation (Supplementary Fig. S3A–C). Notably, increased membrane permeability during SCV stress facilitates prophage release into the extracellular milieu, possibly disseminating phage-encoded virulence factors (Fig. 1F and Supplementary Fig. S3E–G) [22, 38]. However, whether these extracellular phage particles contribute to horizontal gene transfer or trigger host immune responses remains unknown. Moreover, whether this prophage mobilization plays a functional role in adaptation or represents collateral damage remains an open question. Persistent membrane instability during later infection stages (stages 4 and 5) may sustain Dam-mediated control, as unresolved membrane damage can perpetuate stress signals that maintain methylation-dependent regulation.

Our methylome profiling revealed that demethylation events and reversibility of methylation marks were relatively uncommon across the infection cycle, as evident by the limited representation of Class I, II, IV, and V sites (Fig. 2D). Interestingly, although >38 000 GATC motifs were identified at stage 1, no loci were exclusively methylated at this stage (Class I), supporting a bias toward *de novo* methylation acquisition under the SCV condition (Fig. 2A and D). While active replication within the SCV still remains debated, local DNA unwinding in response to stress may expose previously occluded sites to Dam, enabling the methylation. In contrast, methylation patterns established during the SCV phase, particularly at Class VI loci, persisted into later stages (4–5) (Fig. 2D). Although Dam is considered to freely diffuse to target sites, the mechanism underlying its selective activity at stress-responsive loci remains unclear [69]. A prevailing hypothesis is that stress-induced alterations in DNA topology, such as localized unwinding or supercoiling, enhance Dam access to cryptic GATC motifs [70, 71]. Thus, our system may not represent a truly stable heritable mechanism, as altered DNA superhelicity likely drives the accessibility of Dam to the GATC site of the *intA* promoter rather than signal-specific mechanisms. Methylation at 6mA reduces DNA minor groove width and increases helix flexibility, potentially creating structural platforms favorable for the binding of transcription factors (e.g. σ^70^) while inhibiting others (e.g. IHF) [72]. Notably, methylation at the *intA* promoter may also facilitate σ^70^ binding, enhancing transcription by optimizing RNA polymerase docking (Figs 3A and 6E). These topological changes may serve as structural memory elements, enabling the persistent methylation of regulatory regions essential for survival under stress. Our study provides the first functional demonstration of the epigenetic memory mechanism in *Salmonella*, centered on *intA* regulation. Moreover, previous studies have shown that IHF expression remains relatively stable across various growth conditions in *E. coli* [73]. Consistent with this, we detected no significant changes in *ihfA* or *ihfB* expression during infection (Supplementary Fig. S10), suggesting that the sustained transcription of *intA* is not driven by altered IHF levels, but rather by persistent promoter methylation that prevents IHF re-binding. However, some key aspects still need to be clarified: (i) The exact number of bacterial generations during the infection cycle, (ii) Residual Dam/IHF levels during post-SCV stages and their correlation, (iii) Direct assessment of DNA supercoiling dynamics and its impact on Dam–IHF binding during post-SCV stages, (iv) Persistence of stress-induced altered DNA topological states during post-SCV stages and their role in maintaining methylation.

The *ctxAB* operon encoded in the CTX prophage region of *Vibrio cholerae* is repressed by H-NS and activated by ToxT; however, there is no evidence for methylation-based regulation [74]. We found that Dam-mediated methylation blocks the re-binding of IHF to the *intA* promoter following stress-induced dissociation, thereby establishing a durable epigenetic switch. This mechanism enables continued *intA* expression after stress resolution, converting a transient signal into a heritable regulatory system. Among the multiple prophages embedded in the *Salmonella* genome, including Gifsy-1, Gifsy-2, and Fels-1, only ST64B was responsive to Dam-mediated regulation [44]. This specificity is likely attributed to the presence of clustered GATC motifs adjacent to the *int/xis* region and the antirepressor gene *sb42*, which remain methylated across all infection stages (Supplementary Fig. S11). Sequence comparison between IntA and the canonical ST64B integrase revealed partial conservation of 26 residues within the N-terminal DNA-binding domain (Supplementary Fig. S12A). Protein homology modeling further indicated structural conservation of the phage integrase SAM-like domain despite overall divergence in the DNA-binding regions (Supplementary Fig. S12B). In λ integrase systems, core catalytic residues (e.g. Y342, H308, and R311) can compensate for the structural divergence in DNA-binding regions, which in turn may support ST64B recombination [75]. Furthermore, the internal stop codon (TGA) can permit the incorporation of selenocysteine via translational readthrough, potentially generating functional integrase variants under selenium-rich conditions; however, this requires experimental validation [76]. Together, our results indicate that SCV-induced methylation at the *intA* promoter creates a stable transcriptional memory, maintaining gene expression during post-SCV stages and enabling *Salmonella* to convert transient host-derived stress signals into sustained transcriptional programs. This regulatory strategy appears to be evolutionarily tailored to *Salmonella*, where the Dam–IHF switch provides lineage-specific control of integrase expression, while broader conservation of IHF-binding motifs points to a potentially ancestral regulatory architecture involving IHF in related species.

## Supplementary Material

gkaf951_Supplemental_Files

## Data Availability

All strains and plasmids in this study are available from the lead contact upon request. Raw reads for sequencing experiments are available at the NCBI Sequence Read Archive (SMRT-seq for WT stages 1, 3, and 5—SRA accession number: SRR31859143, SRR31859144, and SRR31859145; MeDIP-seq for WT stages 1, 2, 3, and 5—SRA accession numbers: SRR32248253, SRR34628687, SRR32248254, and SRR32248255; MeDIP-seq for *Δdam* stages 1, 2, 3, and 5—SRA accession number: SRR32929628, SRR34628686, SRR32929629, and SRR32929630; International Nucleotide Sequence Database Collaboration *et al.*, 2011). Processed data were deposited into Zenodo with a permanent DOI with two versions (https://doi.org/10.5281/zenodo.15320847 and https://doi.org/10.5281/zenodo.15359496). Custom scripts are available upon request.

## References

[1] Pillay TD, Hettiarachchi SU, Gan J et al. Speaking the host language: how *Salmonella* effector proteins manipulate the host. Microbiology. 2023; 169:001342 10.1099/mic.0.001342. 37279149 PMC10333799

[2] Göser V, Sander N, Schulte M et al. Single molecule analyses reveal dynamics of *Salmonella* translocated effector proteins in host cell endomembranes. Nat Commun. 2023; 14:1240. 36870997 10.1038/s41467-023-36758-9 PMC9985595

[3] Azimi T, Zamirnasta M, Sani MA et al. Molecular mechanisms of *Salmonella* effector proteins: a comprehensive review. IDR. 2020; 13:11–26. 10.2147/IDR.S230604. PMC6954085 32021316

[4] Li M, Tripathi-Giesgen I, Schulman BA et al. *In situ* snapshots along a mammalian selective autophagy pathway. Proc Natl Acad Sci USA. 2023; 120:e2221712120 10.1073/pnas.2221712120. 36917659 PMC10041112

[5] Jennings E, Thurston TLM, Holden DW *Salmonella* SPI-2 type III secretion system effectors: molecular mechanisms and physiological consequences. Cell Host Microbe. 2017; 22:217–31. 10.1016/j.chom.2017.07.009. 28799907

[6] Röder J, Felgner P, Hensel M Comprehensive single cell analyses of the nutritional environment of intracellular *Salmonella enterica*. Front Cell Infect Microbiol. 2021; 11:624650. 33834004 10.3389/fcimb.2021.624650 PMC8021861

[7] Kim JS, Liu L, Kant S et al. Anaerobic respiration of host-derived methionine sulfoxide protects intracellular *Salmonella* from the phagocyte NADPH oxidase. Cell Host Microbe. 2024; 32:411–24. 10.1016/j.chom.2024.01.004. 38307020 PMC11396582

[8] Zhang W, Lyu L, Xu Z et al. Integrative DNA methylome and transcriptome analysis reveals DNA adenine methylation is involved in *Salmonella enterica* Typhimurium response to oxidative stress. Microbiol Spectr. 2023; 11:e0247923 10.1128/spectrum.02479-23. 37882553 PMC10715015

[9] Westphal LL, Sauvey P, Champion MM et al. Genomewide Dam methylation in *Escherichia coli* during long-term stationary phase. Msystems. 2016; 1:e00130–16. 10.1128/mSystems.00130-16. 27981240 PMC5155068

[10] Shell SS, Prestwich EG, Baek SH et al. DNA methylation impacts gene expression and ensures hypoxic survival of *mycobacterium tuberculosis*. PLoS Pathog. 2013; 9:e1003419 10.1371/journal.ppat.1003419. 23853579 PMC3701705

[11] Richards EJ Inherited epigenetic variation-revisiting soft inheritance. Nat Rev Genet. 2006; 7:395–401. 10.1038/nrg1834. 16534512

[12] He L, Wu W, Zinta G et al. A naturally occurring epiallele associates with leaf senescence and local climate adaptation in *Arabidopsis accessions*. Nat Commun. 2018; 9:460 10.1038/s41467-018-02839-3. 29386641 PMC5792623

[13] Sharif J, Koseki H Hemimethylation: DNA’s lasting odd couple. Science. 2018; 359:1102–3. 10.1126/science.aat0789. 29590029

[14] Thomas SL, Xu TH, Carpenter BL et al. DNA strand asymmetry generated by CpG hemimethylation has opposing effects on CTCF binding. Nucleic Acids Res. 2023; 51:5997–6005. 10.1093/nar/gkad293. 37094063 PMC10325916

[15] Hua X, Zhou H, Wu HC et al. Tumor detection by analysis of both symmetric- and hemi-methylation of plasma cell-free DNA. Nat Commun. 2024; 15:6113 10.1038/s41467-024-50471-1. 39030196 PMC11271492

[16] Sánchez-Romero MA, Cota I, Casadesús J DNA methylation in bacteria: from the methyl group to the methylome. Curr Opin Microbiol. 2015; 25:9–16. 10.1016/j.mib.2015.03.004. 25818841

[17] Bergerat A, Kriebardis A, Guschlbauer W Preferential site-specific hemimethylation of GATC sites in pBR322 DNA by Dam methyltransferase from *Escherichia coli*. J Biol Chem. 1989; 264:4064–70. 10.1016/S0021-9258(19)84962-1. 2645286

[18] Anton BP, Roberts RJ Beyond restriction modification: epigenomic roles of DNA methylation in prokaryotes. Annu Rev Microbiol. 2021; 75:129–49. 10.1146/annurev-micro-040521-035040. 34314594

[19] Kwun MJ, Ion AV, Oggioni MR et al. Diverse regulatory pathways modulate bet hedging of competence induction in epigenetically-differentiated phase variants of *Streptococcus pneumoniae*. Nucleic Acids Res. 2023; 51:10375–94. 10.1093/nar/gkad760. 37757859 PMC10602874

[20] Roodsant TJ, van der Putten B, Brizuela J et al. The streptococcal phase-variable type I restriction modification system SsuCC20p dictates the methylome of *Streptococcus suis* impacting the transcriptome and virulence in a zebrafish larvae infection model. mBio. 2024; 15:e0225923 10.1128/mbio.02259-23. 38063379 PMC10790761

[21] Yamazaki Y, Ito T, Nakagawa S et al. Altered genomic methylation promotes *Staphylococcus aureus* persistence in hospital environment. Nat Commun. 2024; 15:9619 10.1038/s41467-024-54033-3. 39511195 PMC11544029

[22] Sargen MR, Helaine S A prophage competition element protects *Salmonella* from lysis. Cell Host Microbe. 2024; 32:2063–79. 10.1016/j.chom.2024.10.012. 39515326 PMC11840918

[23] Yates CR, Nguyen A, Liao J et al. What’s on a prophage: analysis of *Salmonella* spp. Prophages identifies a diverse range of cargo with multiple virulence- and metabolism-associated functions. mSphere. 2024; 9:e0003124 10.1128/msphere.00031-24. 38775467 PMC11332146

[24] Ragunathan PT, Ng Kwan Lim E, Ma X et al. Mechanisms of regulation of cryptic prophage-encoded gene products in *Escherichia coli*. J Bacteriol. 2023; 205:e0012923 10.1128/jb.00129-23. 37439671 PMC10448788

[25] Stone CJ, Boyer GF, Behringer MG Differential adenine methylation analysis reveals increased variability in 6mA in the absence of methyl-directed mismatch repair. mBio. 2023; 14:e0128923 10.1128/mbio.01289-23. 37796009 PMC10653831

[26] Bonnington KE, Kuehn MJ Outer membrane vesicle production facilitates LPS remodeling and outer membrane maintenance in *Salmonella* during environmental transitions. mBio. 2016; 7:e01532–16. 10.1128/mBio.01532-16. 27795394 PMC5082901

[27] Tsai CN, MacNair CR, Cao MPT et al. Targeting two-component systems uncovers a small-molecule inhibitor of *Salmonella* virulence. Cell Chem Biol. 2020; 27:793–805. 10.1016/j.chembiol.2020.04.005. 32413287

[28] Chakraborty S, Mizusaki H, Kenney LJ A FRET-based DNA biosensor tracks OmpR-dependent acidification of *Salmonella* during macrophage infection. PLoS Biol. 2015; 13:e1002116 10.1371/journal.pbio.1002116. 25875623 PMC4397060

[29] McIntosh A, Meikle LM, Ormsby MJ et al. SipA activation of caspase-3 is a decisive mediator of host cell survival at early stages of *Salmonella* enterica Serovar typhimurium infection. Infect Immun. 2017; 85:10 10.1128/IAI.00393-17. PMC5563584 28630067

[30] Datsenko KA, Wanner BL One-step inactivation of chromosomal genes in *Escherichia coli* K-12 using PCR products. Proc Natl Acad Sci USA. 2000; 97:6640–5. 10.1073/pnas.120163297. 10829079 PMC18686

[31] Zhao JP, Zhu H, Guo XP et al. AU-rich long 3' untranslated region regulates gene expression in bacteria. Front Microbiol. 2018; 9:3080 10.3389/fmicb.2018.03080. 30619162 PMC6299119

[32] Han S, Min J, Park Y et al. Fine-tuning regulation of (p)ppGpp-driven outer membrane vesicle formation in *Acinetobacter baumannii*. FEBS J. 2025; 292:3696–717. 10.1111/febs.70087. 40172085

[33] Yang J, Yun S, Park W Blue light sensing BlsA-mediated modulation of meropenem resistance and biofilm formation in *Acinetobacter baumannii*. Msystems. 2023; 8:3696–717. 10.1128/msystems.00897-22. PMC9948694 36622157

[34] Yang J, Son Y, Kang M et al. AamA-mediated epigenetic control of genome-wide gene expression and phenotypic traits in *Acinetobacter baumannii* ATCC 17978. Microb Genom. 2023b; 9:mgen001093. 37589545 10.1099/mgen.0.001093 PMC10483419

[35] Fang G, Munera D, Friedman DI et al. Genome- wide mapping of methylated adenine residues in pathogenic *Escherichia coli* using single-molecule real-time sequencing. Nat Biotechnol. 2012; 30:1232–9. 10.1038/nbt.2432. 23138224 PMC3879109

[36] Flusberg BA, Webster DR, Lee JH et al. Direct detection of DNA methylation during single-molecule, real- time sequencing. Nat Methods. 2010; 7:461–5. 10.1038/nmeth.1459. 20453866 PMC2879396

[37] McIntyre ABR, Alexander N, Grigorev K et al. Single-molecule sequencing detection of N6-methyladenine in microbial reference materials. Nat Commun. 2019; 10:579 10.1038/s41467-019-08289-9. 30718479 PMC6362088

[38] Zhang Y, Liu T, Meyer CA et al. Model-based analysis of ChIP-Seq (MACS). Genome Biol. 2008; 9:R137 10.1186/gb-2008-9-9-r137. 18798982 PMC2592715

[39] Park M, Patel N, Keung AJ et al. Engineering epigenetic regulation using synthetic read-write modules. Cell. 2019; 176:227–238. 10.1016/j.cell.2018.11.002. 30528434 PMC6329643

[40] Murray H, Koh A Multiple regulatory systems coordinate DNA replication with cell growth in *Bacillus subtilis*. PLoS Genet. 2014; 10:e1004731 10.1371/journal.pgen.1004731. 25340815 PMC4207641

[41] Paudel S, Severin GB, Pirani A et al. Multiplexed PCR to measure *in situ* growth rates of uropathogenic *E. coli* during experimental urinary tract infection. Appl Environ Microb. 2025; 91:e02382–24. 10.1128/aem.02382-24. PMC12366353 40742111

[42] Bao Q, Christ N, Dröge P Single-chain integration host factors as probes for high-precision nucleoprotein complex formation. Gene. 2004; 343:99–106. 10.1016/j.gene.2004.08.030. 15563835

[43] Islam F, Mishra PP Molecular insight into the structural dynamics of Holliday junctions modulated by integration host factor. J Phys Chem B. 2024; 128:5642–57. 10.1021/acs.jpcb.4c02997. 38812070

[44] Alonso A, Pucciarelli MG, Figueroa-Bossi N et al. Increased excision of the *Salmonella* prophage ST64B caused by a deficiency in Dam methylase. J Bacteriol. 2005; 187:7901–11. 10.1128/JB.187.23.7901-7911.2005. 16291663 PMC1291290

[45] Sullivan GJ, Barquist L, Cain AK A method to correct for local alterations in DNA copy number that bias functional genomics assays applied to antibiotic-treated bacteria. Msystems. 2024; 9:e0066523 10.1128/msystems.00665-23. 38470252 PMC11019837

[46] Son Y, Kim B, Kim P et al. Unexpected vulnerability of *Enterococcus faecium* to polymyxin B under anaerobic condition. Gut Microbes. 2024; 16:2438465 10.1080/19490976.2024.2438465. 39663231 PMC11651277

[47] Son Y, Min J, Jang I et al. Enhanced mechanical properties of living and regenerative building materials by filamentous *Leptolyngbya boryana*. Cell Rep Phys Sci. 2024; 5:102098 10.1016/j.xcrp.2024.102098.

[48] Barakat M, Ortet P, Whitworth DE P2RP: a web-based framework for the identification and analysis of regulatory proteins in prokaryotic genomes. BMC Genomics. 2013; 14:269 10.1186/1471-2164-14-269. 23601859 PMC3637814

[49] Cassiano MHA, Silva-Rocha R Benchmarking bacterial promoter prediction tools: potentialities and limitations. Msystems. 2020; 5:e00439–20. 10.1128/mSystems.00439-20. 32843538 PMC7449607

[50] Vallenet D, Calteau A, Dubois M et al. MicroScope: an integrated platform for the annotation and exploration of microbial gene functions through genomic, pangenomic and metabolic comparative analysis. Nucleic Acids Res. 2020; 48:D579–D589. 31647104 10.1093/nar/gkz926 PMC7145621

[51] Karash S, Liyanage R, Qassab A et al. A comprehensive assessment of the genetic determinants in *Salmonella* Typhimurium for resistance to hydrogen peroxide using proteogenomics. Sci Rep. 2017; 7:17073 10.1038/s41598-017-17149-9. 29213059 PMC5719062

[52] Schroeder N, Mota LJ, Méresse S *Salmonella*-induced tubular networks. Trends Microbiol. 2011; 19:268–77. 10.1016/j.tim.2011.01.006. 21353564

[53] Uppalapati S, Kant S, Liu L et al. Prophage terminase with tRNase activity sensitizes *Salmonella enterica* to oxidative stress. Science. 2024; 384:100–5. 10.1126/science.adl3222. 38574144 PMC11443816

[54] Justice SS, Hunstad DA, Cegelski L et al. Morphological plasticity as a bacterial survival strategy. Nat Rev Micro. 2008; 6:162–8. 10.1038/nrmicro1820. 18157153

[55] Zhang D, Yin F, Qin Q et al. Molecular responses during bacterial filamentation reveal inhibition methods of drug-resistant bacteria. Proc Natl Acad Sci USA. 2023; 120:e2301170120 10.1073/pnas.2301170120. 37364094 PMC10318954

[56] Davey HM, Hexley P Red but not dead? Membranes of stressed *saccharomyces cerevisiae* are permeable to propidium iodide. Environ Microbiol. 2011; 13:163–71. 10.1111/j.1462-2920.2010.02317.x. 21199254

[57] Rosenberg M, Azevedo NF, Ivask A Propidium iodide staining underestimates viability of adherent bacterial cells. Sci Rep. 2019; 9:6483 10.1038/s41598-019-42906-3. 31019274 PMC6482146

[58] Stephenson SA, Brown PD Epigenetic influence of dam methylation on gene expression and attachment in uropathogenic *Escherichia coli*. Front Public Health. 2016; 4:131 10.3389/fpubh.2016.00131. 27446897 PMC4921776

[59] Rice PA, Yang S, Mizuuchi K et al. Crystal structure of an IHF-DNA complex: a protein-induced DNA U-turn. Cell. 1996; 87:1295–306. 10.1016/S0092-8674(00)81824-3. 8980235

[60] Hustmyer CM, Landick R Bacterial chromatin proteins, transcription, and DNA topology: inseparable partners in the control of gene expression. Mol Microbiol. 2024; 122:81–112. 10.1111/mmi.15283. 38847475 PMC11260248

[61] Lee SY, Lim CJ, Dröge P et al. Regulation of bacterial DNA packaging in early stationary phase by competitive DNA binding of Dps and IHF. Sci Rep. 2015; 5:18146 10.1038/srep18146. 26657062 PMC4677351

[62] Romero Romero ML, Poehls J, Kirilenko A et al. Environment modulates protein heterogeneity through transcriptional and translational stop codon readthrough. Nat Commun. 2024; 15:4446 10.1038/s41467-024-48387-x. 38789441 PMC11126739

[63] Toledano I, Supek F, Lehner B Genome-scale quantification and prediction of pathogenic stop codon readthrough by small molecules. Nat Genet. 2024; 56:1914–24. 10.1038/s41588-024-01878-5. 39174735 PMC11387191

[64] Debatisse K, Lopez P, Poli M et al. Redefining the bacteriophage mv4 site-specific recombination system and the sequence specificity of its *attB* and core-*attP* sites. Mol Microbiol. 2024; 121:1200–16. 10.1111/mmi.15275. 38705589

[65] Sewitz S, Crellin P, Chalmers R The positive and negative regulation of Tn10 transposition by IHF is mediated by structurally asymmetric transposon arms. Nucleic Acids Res. 2003; 31:5868–76. 10.1093/nar/gkg797. 14530435 PMC219475

[66] Park JY, Jang M, Lee SM et al. Unveiling the novel regulatory roles of RpoD-family sigma factors in *Salmonella* Typhimurium heat shock response through systems biology approaches. PLoS Genet. 2024; 20:e1011464 10.1371/journal.pgen.1011464. 39471211 PMC11548764

[67] Stokar-Avihail A, Fedorenko T, Hör J et al. Discovery of phage determinants that confer sensitivity to bacterial immune systems. Cell. 2023; 186:1863–76. 10.1016/j.cell.2023.02.029. 37030292

[68] Ruan Y, Tang H, Cai T et al. Efficient genetic manipulation of *Shewanella* through targeting defense islands. Appl Environ Microb. 2025; 91:e0249924 10.1128/aem.02499-24. PMC12016545 40116498

[69] Urig S, Gowher H, Hermann A et al. The *Escherichia coli* Dam DNA methyltransferase modifies DNA in a highly processive reaction. J Mol Biol. 2002; 319:1085–96. 10.1016/S0022-2836(02)00371-6. 12079349

[70] Dorman CJ DNA supercoiling and transcription in bacteria: a two-way street. BMC Mol and Cell Biol. 2019; 20:26 10.1186/s12860-019-0211-6. 31319794 PMC6639932

[71] Dash S, Palma CSD, Baptista ISC et al. Alteration of DNA supercoiling serves as a trigger of short-term cold shock repressed genes of *E. coli*. Nucleic Acids Res. 2022; 50:8512–28. 10.1093/nar/gkac643. 35920318 PMC9410904

[72] Zhou J, Horton JR, Blumenthal RM et al. *Clostridioides difficile* specific DNA adenine methyltransferase CamA squeezes and flips adenine out of DNA helix. Nat Commun. 2021; 12:3436 10.1038/s41467-021-23693-w. 34103525 PMC8187626

[73] Kasho K, Oshima T, Chumsakul O et al. Whole-genome analysis reveals that the nucleoid protein IHF predominantly binds to the replication origin *oriC* specifically at the time of initiation. Front Microbiol. 2021; 12:697712 10.3389/fmicb.2021.697712. 34475859 PMC8407004

[74] Stonehouse EA, Hulbert RR, Nye MB et al. H-NS binding and repression of the ctx promoter in *Vibrio cholerae*. J Bacteriol. 2011; 193:979–88. 10.1128/JB.01343-09. 21169492 PMC3028689

[75] Williams JD, Voziyanova E, Voziyanov Y The bacteriophage lambda integrase catalytic domain can be modified to act with the regulatory domain as a recombination-competent binary recombinase. J Biol Chem. 2023; 299:102721 10.1016/j.jbc.2022.102721. 36410432 PMC9791396

[76] Liu Z, Wang J, Shi Y et al. Seryl-tRNA synthetase promotes translational readthrough by mRNA binding and involvement of the selenocysteine incorporation machinery. Nucleic Acids Res. 2023; 51:10768–81. 10.1093/nar/gkad773. 37739431 PMC10602924

