# Supplementary material for: Dam-dependent epigenetic memory regulates prophage reintegration in Salmonella
Source: Nucleic Acids Res. 2025 Sep 23;53(18):gkaf951. doi: 10.1093/nar/gkaf951 (PMC12455584; doi:10.1093/nar/gkaf951)
Supplement: gkaf951_Supplemental_Files [file gkaf951_supplemental_files.zip › Figures S1-S12.pdf]

Dam-dependent transcriptional memory regulates prophage reintegration in *Salmonella*

Jihye Yang, Yongjun Son, Jinwon Park and Woojun Park*

Laboratory of Molecular Environmental Microbiology, Department of Environmental Science and Ecological Engineering, Korea University, Seoul, 02841, Republic of Korea.

Keywords: *Salmonella*, *Salmonella*-containing-vacuole, Epigenetics, Methylome, Prophage, Integrase

Running title: Transcriptional memory during *Salmonella* pathogenesis

*__Corresponding author:__
Dr. Woojun Park, Department of Environmental Science and Ecological Engineering, Korea

University, Seoul, 02841, Republic of Korea
E-mail: wpark@korea.ac.kr
Fax: +82-2-953-0737 / Phone: +82-2-3290-3067

Supplementary Figure 1.

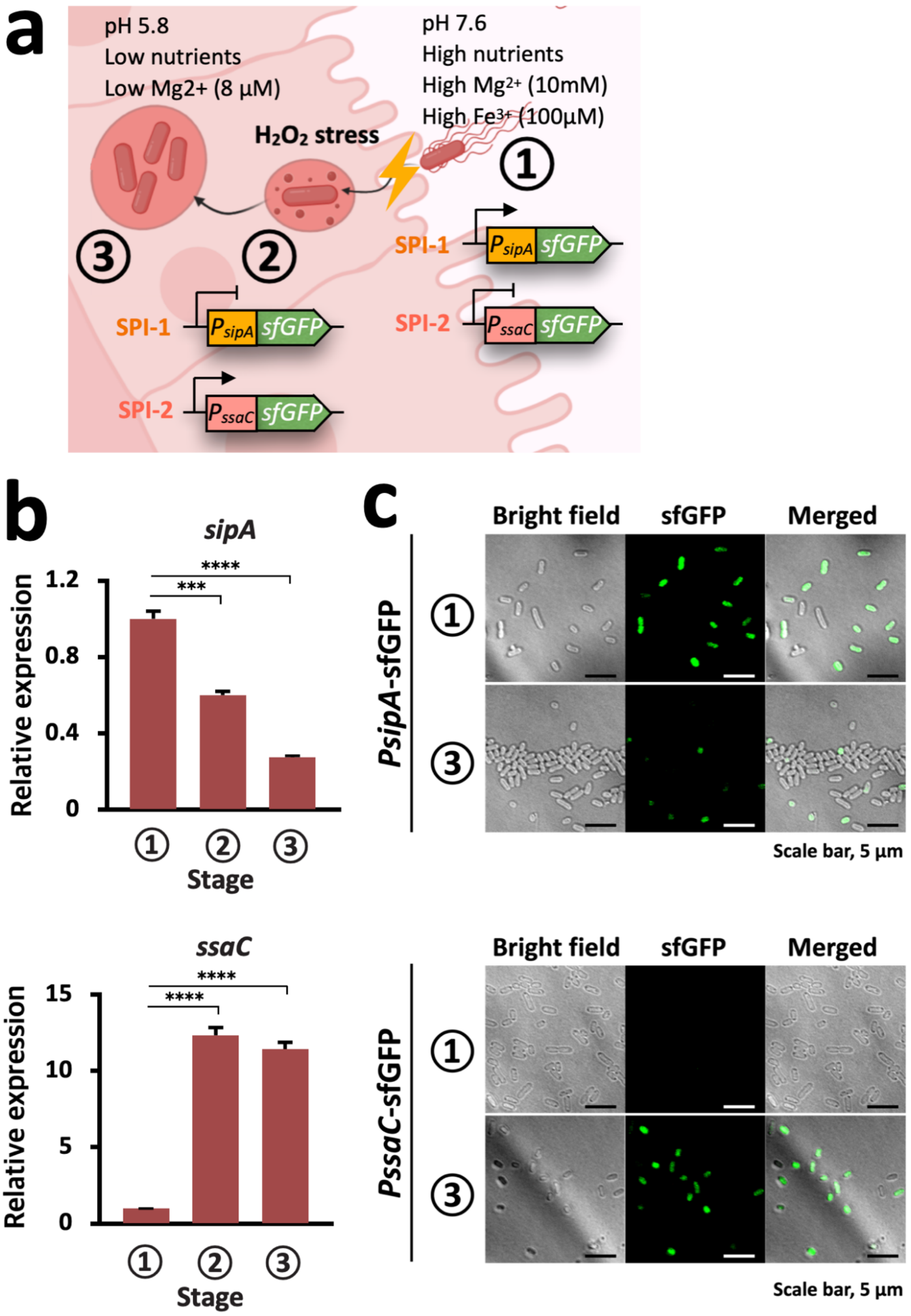

Supplementary Figure 1. Validation of the in vitro model using virulence gene expression. (a) Schematic representation of the *in vitro* infection model. (b) Relative mRNA expression levels of *sipA* and *ssaC* at different stress stages, as measured by qRT-PCR. Values are normalized to stage 1. Error bars represent mean ± SD of biological triplicates. Statistical significance was determined byStudent's *t*-test (two-tailed) for comparisons between two groups (***$p < 0.001$ and ****$p < 0.0001$). (c) Confocal laser scanning microscopy (CLSM) analyses of *PsipA*-sfGFP and *PssaC*-sfGFP transcriptional reporters at stage 1 and stage 3. Representative images are shown for bright fields, sfGFP, and merged channels. Scale bars, 5 µm.

Supplementary Figure 2.

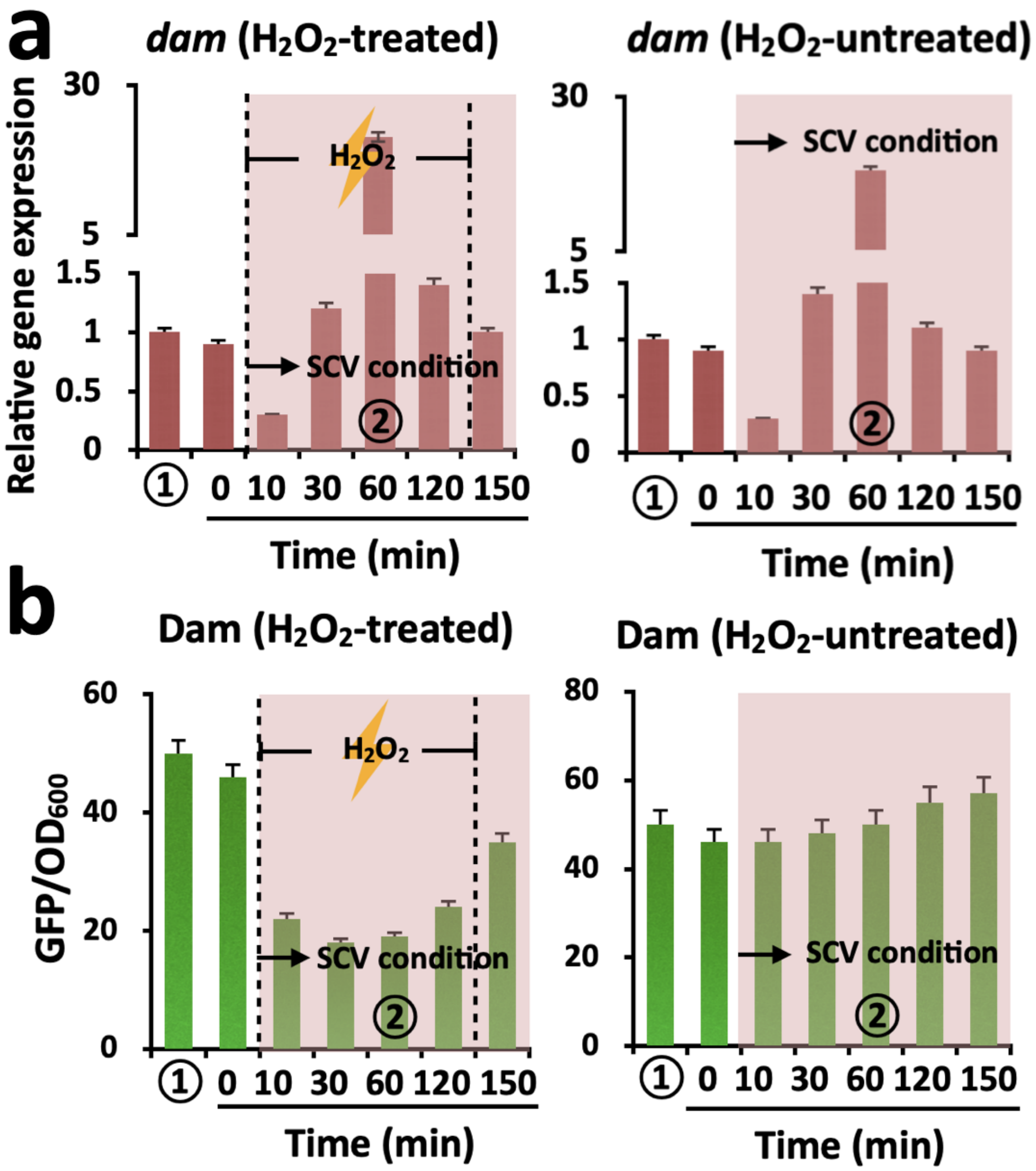

Supplementary Figure 2. Relative *dam* transcription and Dam-msfGFP level during stage 1~2, with or without H_2O_2. (a) qRT-PCR of *dam* mRNA with (left) or without (right) H_2O_2. (b) Fluorescence detection of Dam-msfGFP with (left) or without (right) H_2O_2. All experiments were performed triplicate.

Supplementary Figure 3.

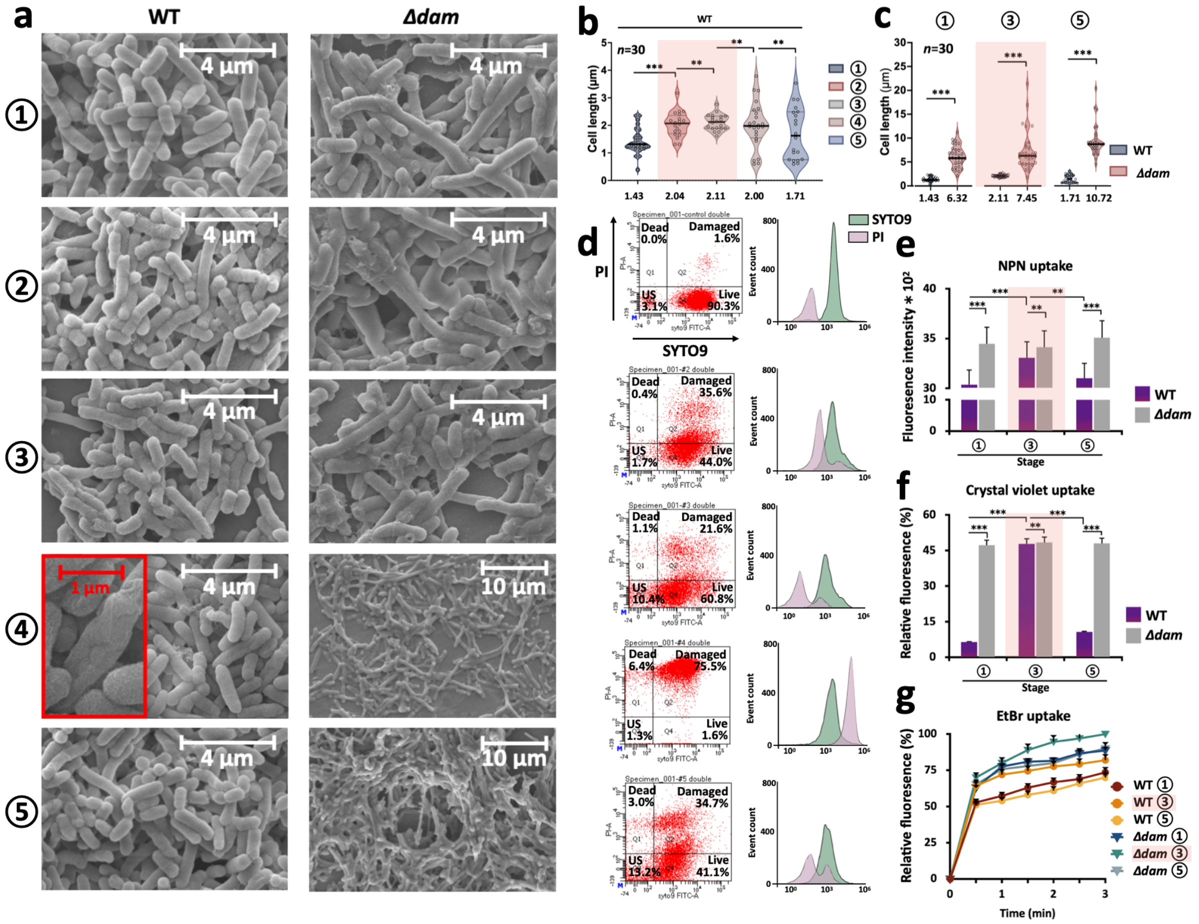

Supplementary Figure 3. Morphological and membrane permeability assays of *S.* Typhimurium 14028s wild-type (WT) and Δ*dam* mutant. (a) Field-emission scanning electron microscopy (FE-SEM) analyses during infection stages (1-5). (b) Length of WT cells (n=30) during the stages (1-5). (c) Length of WT and Δ*dam* cells (n=30, respectively) during stages 1, 3, and 5. All measurements were performed using the ImageJ software (National Institutes of Health, USA). (d) Membrane permeability assays using (e) 1-N-phenylnaphthylamine (NPN), (f) crystal violet, and (g) ethidium bromide (EtBr). All experiments were performed triplicate. *P*-values are designated by asterisks (***p* < 0.01, ****p* < 0.001, *****p* < 0.0001 based on Student's *t*-test, two-tailed. ns, not specific).

Supplementary Figure 4.

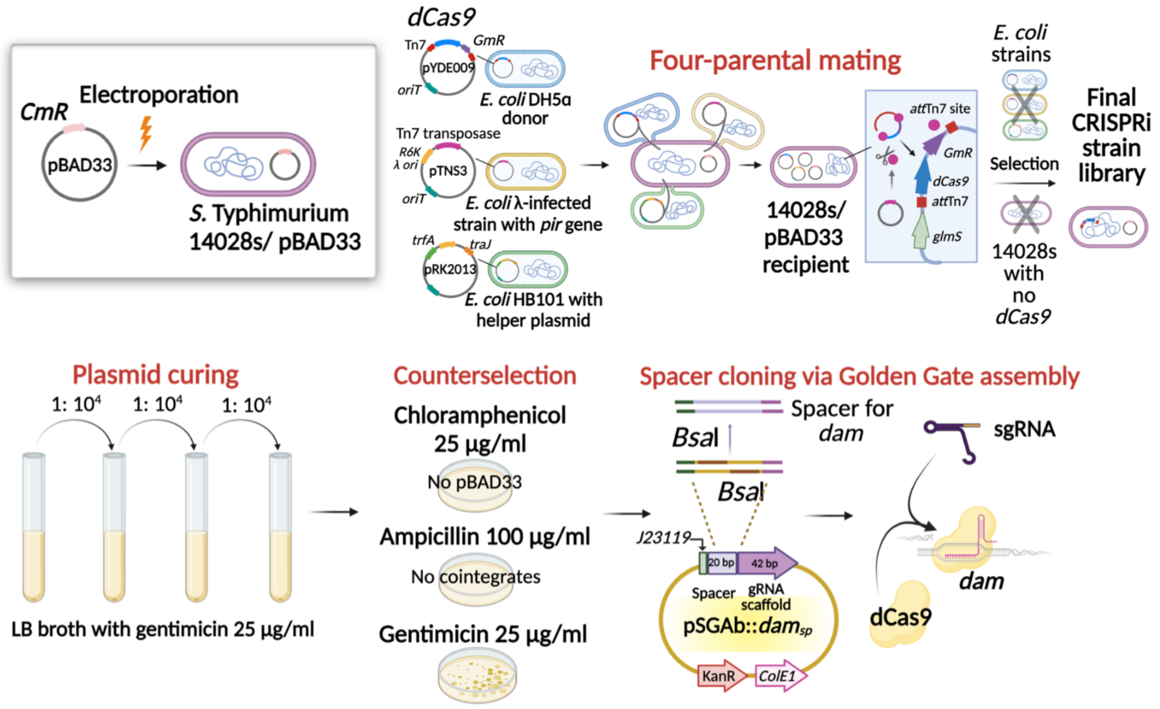

Supplementary Figure 4. CRISPR interference (CRISPRi) system construction. The mini-Tn7 element of the pYDE009, which contains an anhydrotetracycline (aTc)-induced CRISPRi module, was introduced into WT strain harboring pBAD33 (14028s/ pBAD33) via four-parental mating. Recipient 14028s/ pBAD33, *E. coli* DH5α (each containing pYDE009 and pTNS3 plasmids), and the helper strain *E. coli* HB101 containing the pRK2013 plasmid were grown overnight at 37 °C with shaking. Each culture (1 mL) was combined to make a 4 mL culture mix, and the sample was pelleted by centrifugation at 7,600 × g for 1 min at 4 °C. The cells were washed with 10 mM $MgSO_4$ and subsequently washed with phosphate buffer saline (PBS). The mixture (500 µL) of the four strains was then plated onto a 0.45-µm pore 47 mm filter on Luria-Bertani (LB) plate and incubated for 4 h at 37 °C. The resulting cells were washed off the filter with 1 mL $MgSO_4$ and 100 µL were spread onto a LB plate containing chloramphenicol (CM) and gentamicin for selection. Integration of *dCas9* at *att*Tn7 site was verified by PCR. Plasmid curing of pBAD33 achieved through serial subculturing without antibiotic selection. Counterselection was performed at LB plates with or without CM. For sgRNA expression, the spacer of the *dam* was cloned to the pSGAb vector via the Golden Gate Assembly and transformed into the chromosomal dCas9-harboring 14028s strain [31].

Supplementary Figure 5.

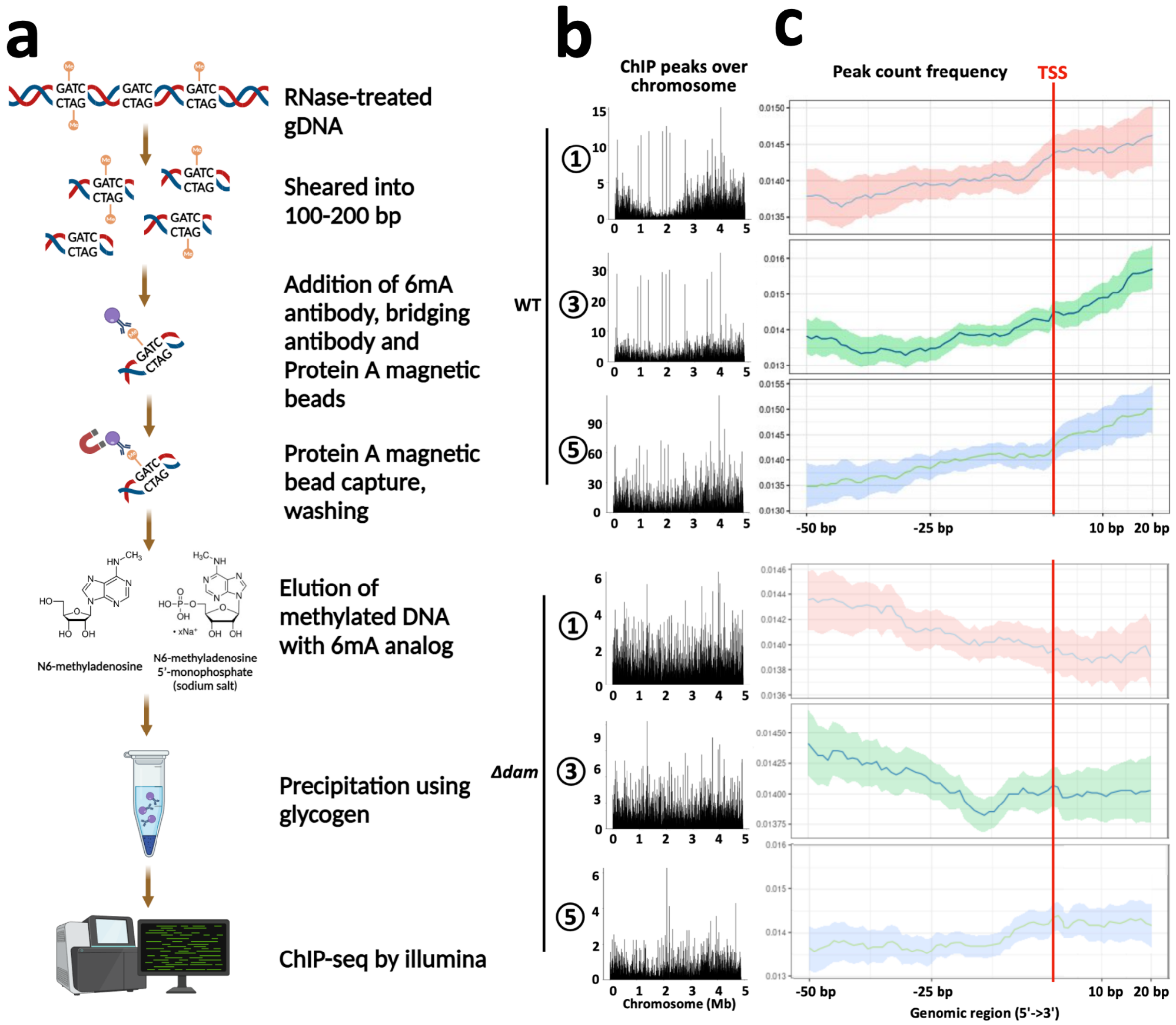

Supplementary Figure 5. Validation of methylated DNA immunoprecipitation sequencing (MeDIP-seq). (a) Schematic overview of procedures performing 6mA immunoprecipitation. (b) ChIP peaks of WT (upper) and Δ*dam* (below) mapped over *S.* Typhimurium 14028s chromosome. (c) ChIP peaks distribution of gene regulatory regions in WT (upper) and Δ*dam* (below) genome. TSS, transcription start site.

Supplementary Figure 6.

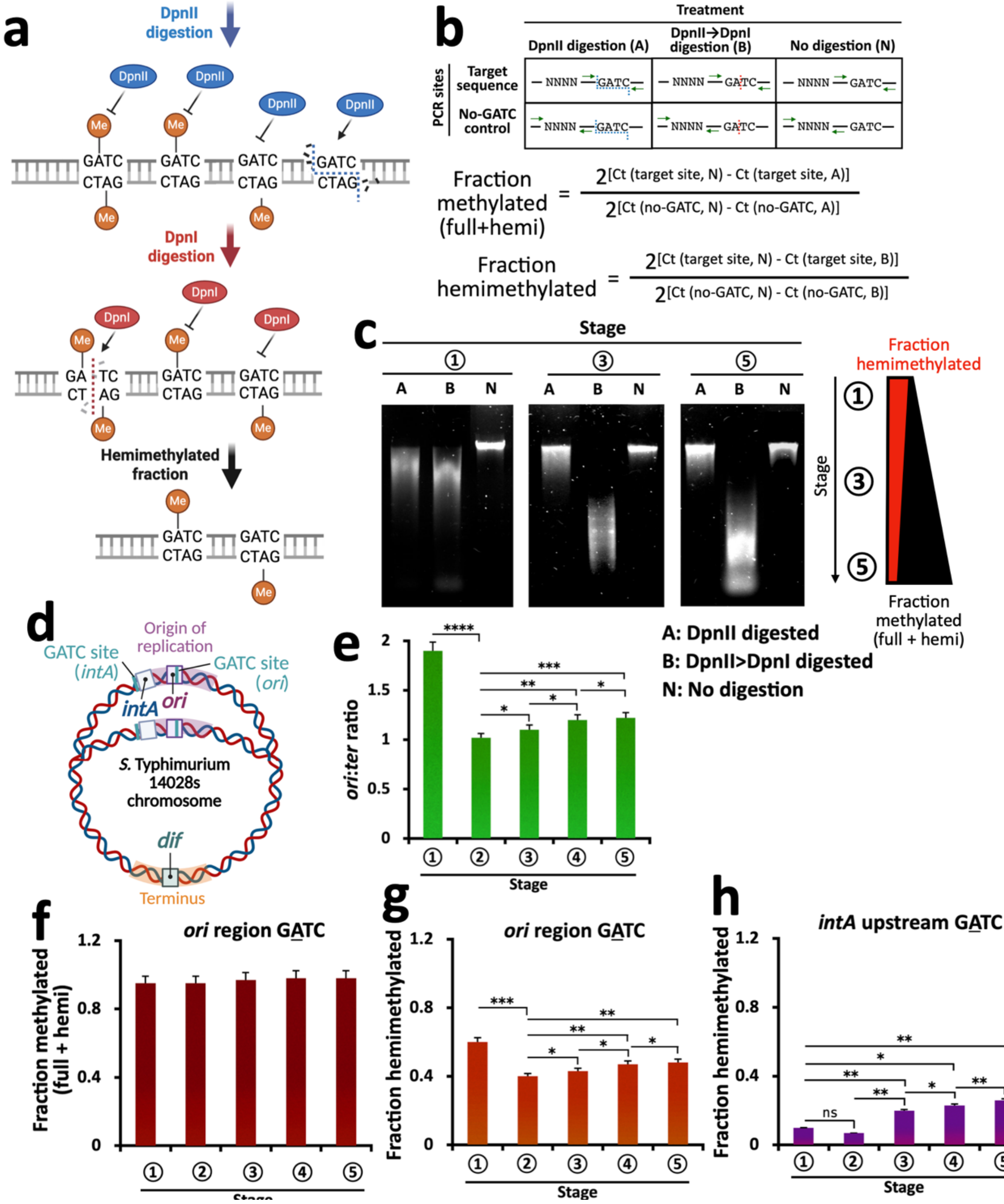

Supplementary Figure 6. Detection of replication-derived hemimethylation levels. (a) Assay schematic. Genomic DNA was subjected to DpnII (cleaves unmethylated GATC) and then to DpnI (cleaves fully methylated G<u>A</u>TC). Templates that remain intact after the sequential digestion are hemimethylated and are selectively amplified by qPCR. (b) Calculation framework. (c) Agarose gels confirming digestion patterns for representative samples from stages 1, 3, and 5. (d) Map of the S*. Typhimurium* 14028s chromosome showing the *ori* and *dif* (terminus) regions used for *ori:ter* MFA and the assayed GATC sites at *ori* and upstream of *intA*. (e) qPCR-based *ori:ter* ratios across stages 1–5. (f) Total methylated fraction at the *ori*-region G<u>A</u>TC. (g) Hemimethylated fraction at the *ori*-region G<u>A</u>TC. No statistically significant differences were detected. (h) Hemimethylated fraction at the *intA* upstream G<u>A</u>TC. All experiments were performed triplicate. *P*-values are designated by asterisks (***p* < 0.01, ****p* < 0.001, *****p* < 0.0001 based on Student's *t*-test, two-tailed. ns, not specific).

Supplementary Figure 7.

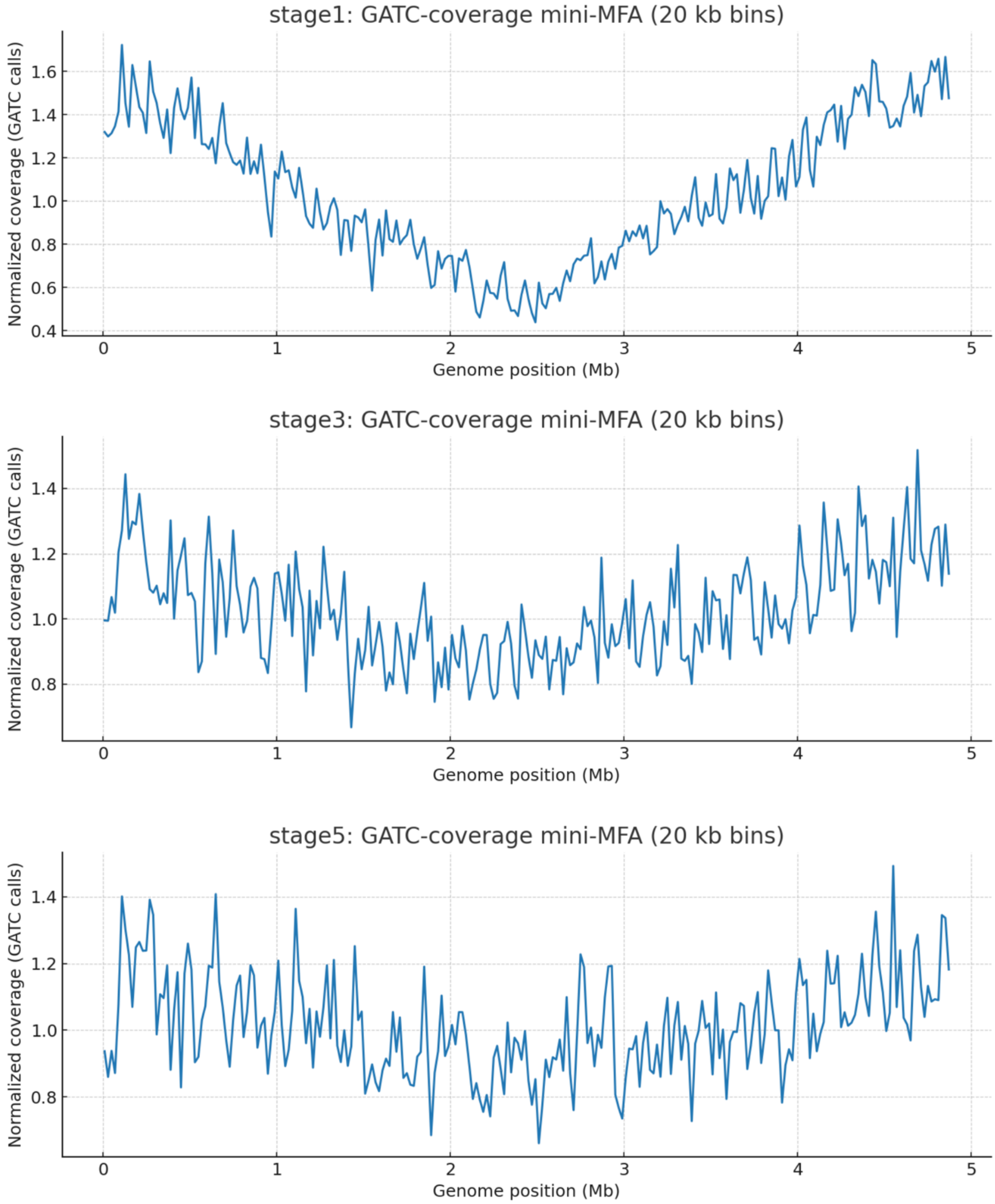

Supplementary Figure 7. Genome-wide replication profiles (mini-MFA) across stages. SMRT-seq coverage was summarized in non-overlapping 20-kb bins across the chromosome. For each bin, normalized coverage was defined as the number of GATC regions covered by primary reads, irrespective of methylation status, divided by the library-wide total of GATC-coverage counts. Line traces show the binned profiles for stage 1, stage 3, and stage 5. Summary *ori:ter* (coverage) values were ≈2.6 (stage 1) and ≈1.1 (stages 3 and 5).

Supplementary Figure 8.

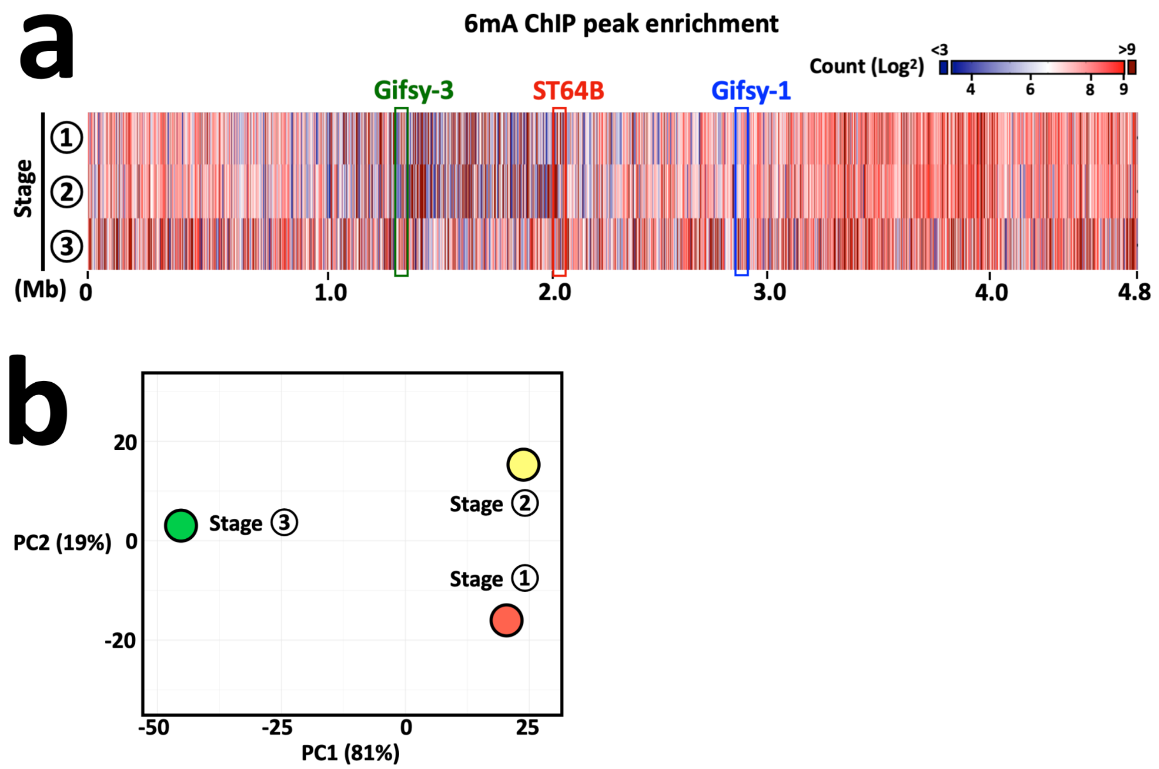

Supplementary Figure 8. 6mA ChIP peaks of 14028s WT strain during stages 1, 2, and 3. (a) Heatmap of 6mA ChIP peak distribution. Genomic DNA was extracted at each stage and fragmented to 100–200 bp. Methylated DNA was immunoprecipitated using a 6mA-specific antibody and sequenced (100 bp paired-end reads, NovaSeq 6000). Low-quality reads and adapters were removed using fastp v0.23.1, and clean reads were aligned to the reference genome (GenBank: CP001363.1) using bowtie2 v2.3.4.3. Heatmap visualization (log_2 normalized counts) shows that the ChIP profile at stage 2 closely mirrors that of stage 1, with broad methylation gain appearing only at stage 3. Colored boxes highlight prophage regions: Gifsy-3 (green), ST64B (red), and Gifsy-1 (blue). (b) Principal component analysis (PCA) of ChIP-seq signals of ChIP peak signals. The PCA plot was visualized using Clustvis 2.0.

Supplementary Figure 9.

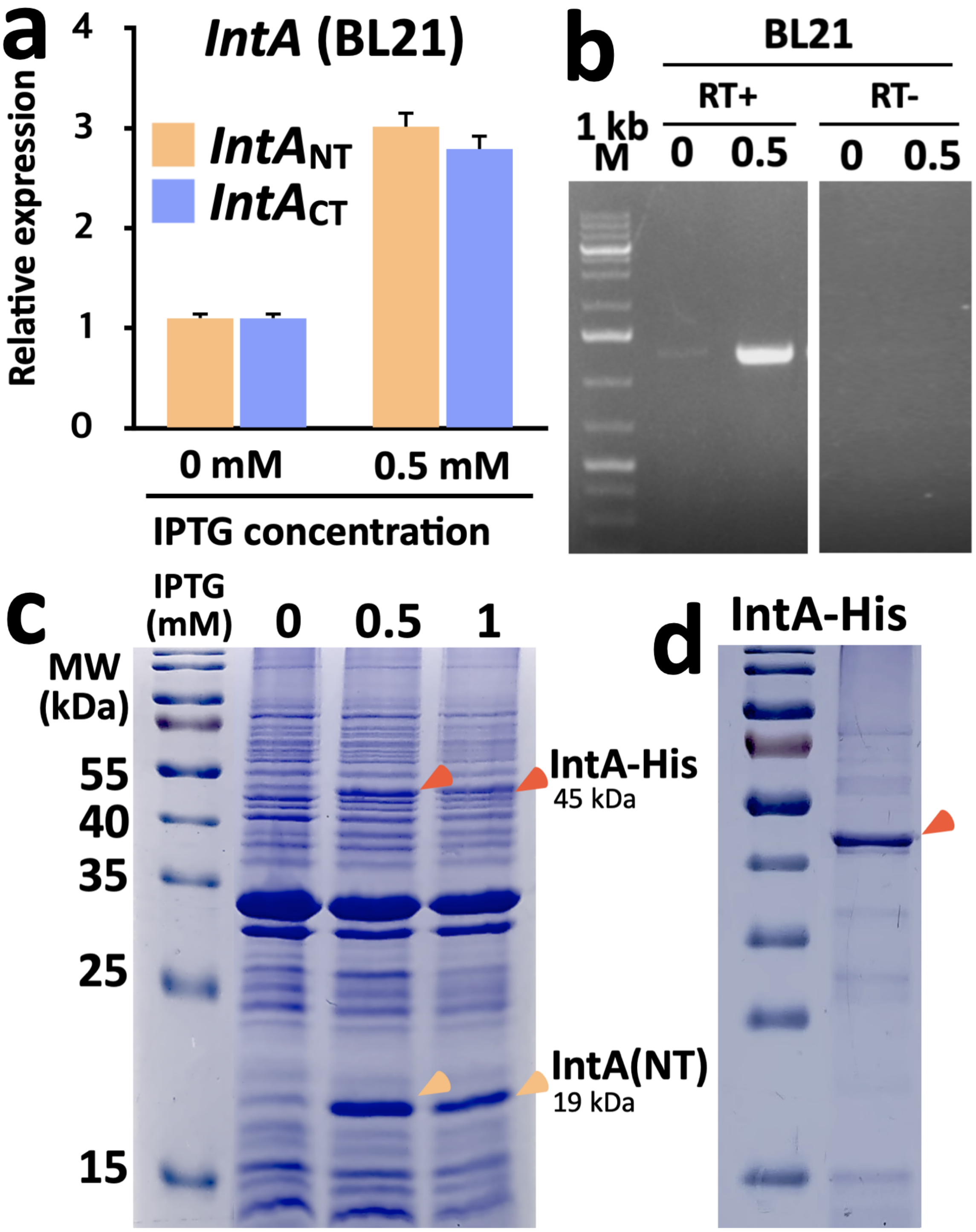

Supplementary Figure 9. Expression of IntA-His in *E. coli* BL21. (a) Isopropyl β-D-1-thiogalactopyranoside (IPTG, 0.5 mM)-induction of *intA* verified by qRT-PCR using primers detecting each N/C-terminal domain. (b) Reverse transcription of *intA* using primers detecting the whole sequence, with (RT+) or without (RT-) the reverse transcriptase. (c) Coomassie stained crude extract of IntA-His expressing BL21. Full-length IntA-His (45 kDa) and C-terminally truncated form [IntA(NT), 19 kDa] is indicated in red and yellow cones, respectively. (d) Purified IntA-His via Ni-NTA column from cytosolic fraction of IPTG-induced (0.5 mM) BL21 cells.

Supplementary Figure 10.

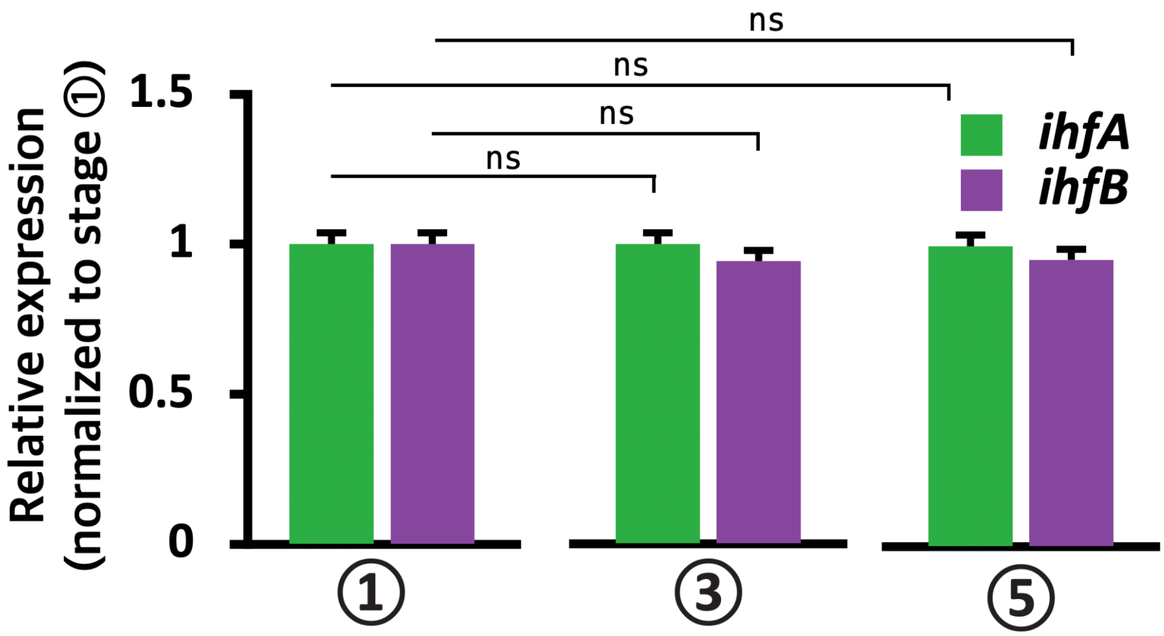

Supplementary Figure 10. Relative expression of *ihfA* and *ihfB* during stages 1, 3, and 5. qRT-PCR was performed using RNA isolated from 14028s WT cells harvested at each indicated stage. Expression levels were normalized to 16S rRNA and are presented relative to stage 1 (set as 1.0). Data represent the mean ± SEM from three independent biological replicates. No significant differences were observed across stages, indicating stable expression of IHF subunits during infection progression.

Supplementary Figure 11.

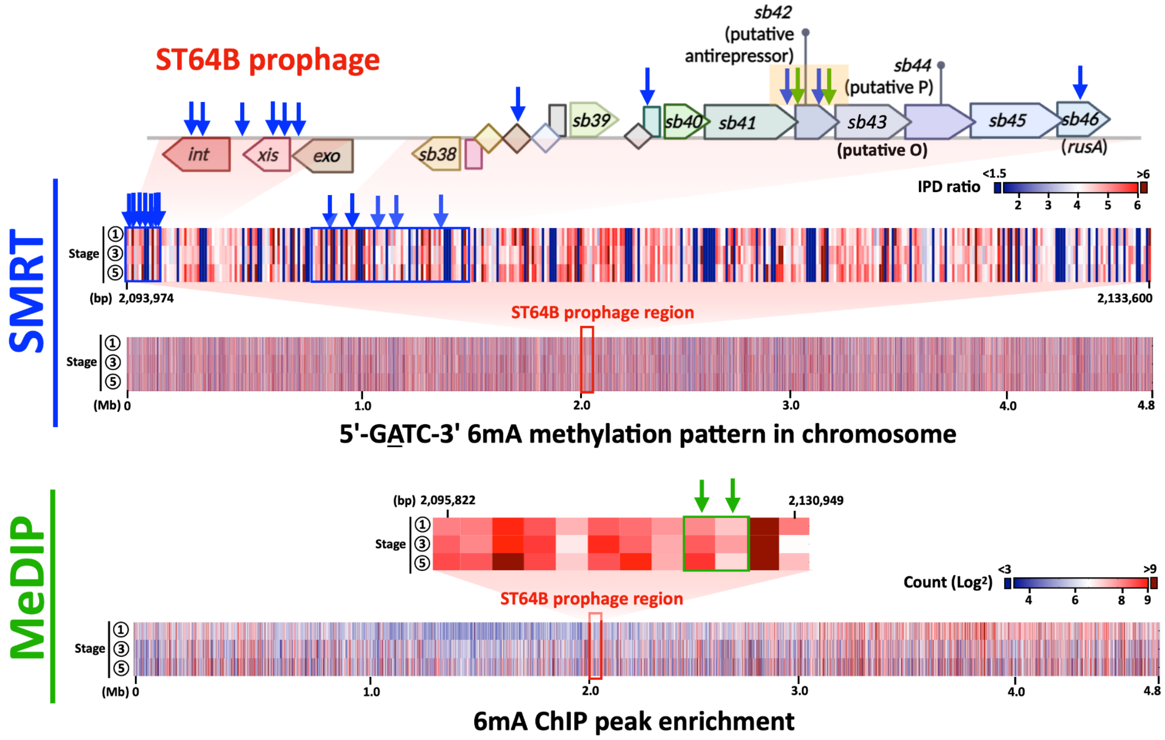

Supplementary Figure 11. Stage-specific 6mA methylation patterns are clustered across the 14028s chromosome. The ST64B prophage region is highlighted in red boxes and magnified above each corresponding chromosomal heatmap. Methylated regions near the *int/xis* and replication regions are indicated in arrows (blue, detected based on SMRT-seq; green, detected based-on MeDIP-seq). Heatmaps were visualized using GraphPad Prism 9.1.3 (Graph-Pad Software).

Supplementary Figure 12.

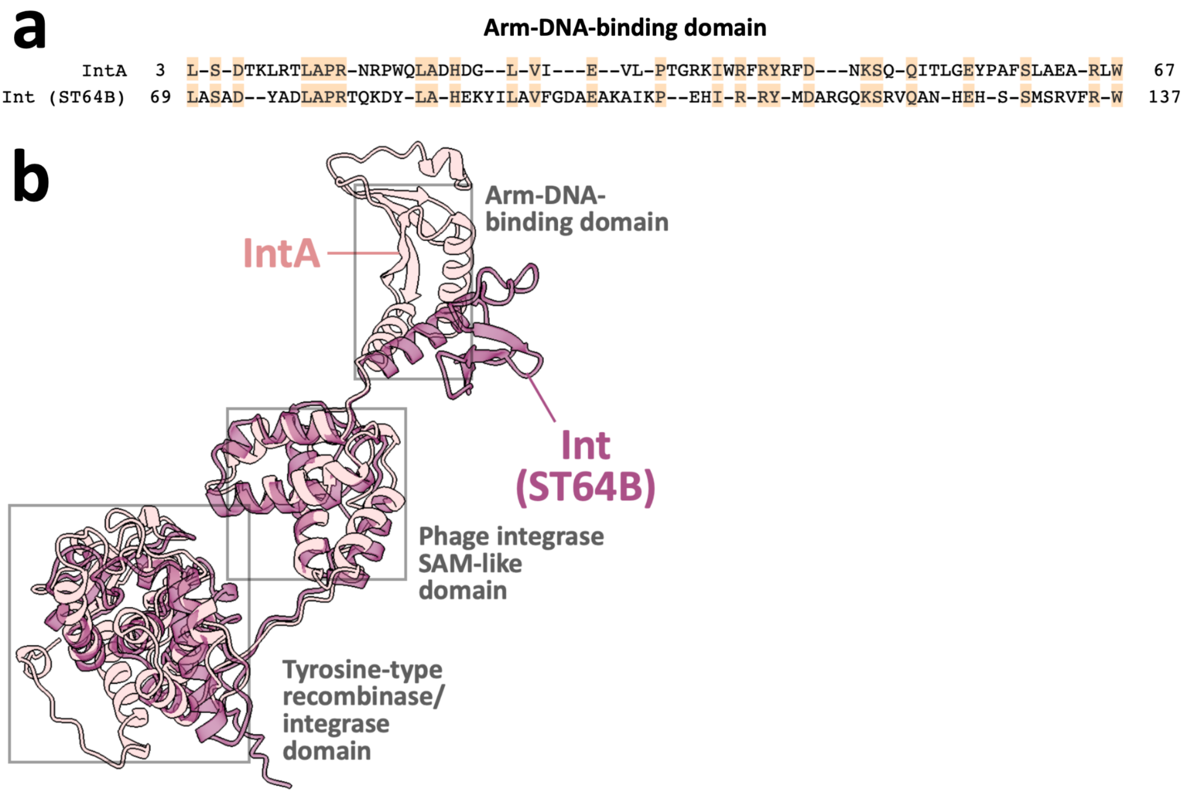

Supplementary Figure 12. Sequence/ homology structural analyses of IntA and ST64B integrase. (a) Sequence alignment of the arm-DNA-binding domain of IntA and ST64B integrase. (b) Structural alignment of IntA and ST64B integrase. Homology models were constructed using AlphaFold3 visualized with ChimeraX. Each domain is indicated in gray boxes. The internal stop codon of IntA is considered as tryptophan (W165), according to the previous studies [62, 63].
